# Poly(ADP-ribose) polymerase 1 orchestrates vascular smooth muscle cell homeostasis in arterial disease

**DOI:** 10.1038/s12276-025-01501-5

**Published:** 2025-08-01

**Authors:** Wenjing Xu, Yichen Wu, Ruiqi Mao, Yujie Jia, Hao Jiang, Fengxiao Zhang, Dan Huang, Ximiao He, Cheng Wang, Kai Huang

**Affiliations:** 1 Clinic Center of Human Gene Research, Union Hospital, Tongji Medical College, Huazhong University of Science and Technology, Wuhan, China; 2 Department of Cardiology, Union Hospital, Tongji Medical College, Huazhong University of Science and Technology, Wuhan, China; 3 Hubei Key Laboratory of Metabolic Abnormalities and Vascular Aging, Wuhan, China; 4 Hubei Clinical Research Center for Metabolic and Cardiovascular Disease, Wuhan, China; 5 Department of Rheumatology, Union Hospital, Tongji Medical College, Huazhong University of Science and Technology, Wuhan, China; 6 School of Basic Medicine, Tongji Medical College, Huazhong University of Science and Technology, Wuhan, China

**Keywords:** Translational research, PolyADP-ribosylation

## Abstract

Smooth muscle cells are remarkably plastic. Their reversible differentiation is required for growth and wound healing but also contributes to pathologies such as atherosclerosis and restenosis. Here we demonstrate the role of poly(ADP-ribose) polymerase 1 (PARP1) as a critical master regulator of vascular smooth muscle cells (VSMC) plasticity. A robust activation of PARP1 in VSMCs was observed in artery stenosis and atherosclerotic plaques of rodents and human. Inhibition or deletion of PARP1 suppressed the VSMC phenotype switch in vivo and in vitro. Further analysis identified myocardin and myocardin-associated serum response factor as substrates of PARP1-mediated poly(ADP-ribosyl)ation reaction. Poly(ADP-ribosyl)ation of myocardin and serum response factor dissociated the complex from CArG motif in the target promoter and then transcriptionally suppressed contractile protein expression. Moreover, we demonstrated that c-Jun mediated the stimulation of VSMC proliferation and migration by PARP1. Notably, interaction with myocardin is an important mechanism repressing c-Jun transcriptional activity in VSMCs. Poly(ADP-ribosyl)ation of myocardin and c-Jun disrupted myocardin–c-Jun interaction and abolished this repression to promote c-Jun transactivation and target gene expression, thus stimulating VSMC proliferation and migration. Our data reveal that activation of PARP1 not only suppresses contractile status but also promotes the synthetic proliferative phenotype of VSMCs, indicating a pivotal role for PARP1 in determining the phenotype of VSMCs. Targeting PARP1 may hold therapeutic potential for vascular pathologies.

## PARP1 regulates vascular smooth muscle cell plasticity

Vascular smooth muscle cells (VSMCs) help to control blood vessel function and can change from a contractile state to a synthetic state, which is important for healing but can also lead to diseases such as atherosclerosis. This study explores how PARP1 affects this change in VSMCs. Researchers used animal models and human samples to study the role of PARP1. They found that PARP1 is highly active in VSMCs during artery injury and disease. By activating PARP1, they observed changes in VSMC behavior, such as increased or decreased cell growth and movement. PARP1 modifies proteins such as myocardin, affecting their ability to control VSMC functions. This modification disrupts the normal suppression of another protein, c-Jun, leading to more cell growth and movement. The study concludes that targeting PARP1 could be a new way to treat vascular diseases by controlling VSMC behavior.

This summary was initially drafted using artificial intelligence, then revised and fact-checked by the author.

## Introduction

Vascular smooth muscle cells (VSMCs) normally express a unique repertoire of contractile proteins, ion channels and signaling molecules required for the modulation of vascular tone. However, upon environmental cues, such as growth factors/cytokines, injury stimuli and mechanical force, VSMCs are capable of switching from a contractile (quiescent, differentiated) status to a synthetic (proliferative, dedifferentiated) phenotype, exhibiting decreased contractility and related proteins expression but enhanced proliferation, migration and synthesis of extracellular matrix components and cytokines^1^. Although phenotypic plasticity of VSMCs is important for vascular development and repair, deregulation of the phenotype switch is an important mechanism driving the onset and progression of neointimal hyperplasia and atherosclerosis, in which VSMCs and VSMC-driven cells constitute the major cell component of the lesions^1–3^. Therefore, clarifying the regulatory mechanism of VSMC phenotype (in particular, identifying factors that promote VSMC phenotype switching) is crucial for developing novel therapeutic approaches against vascular diseases such as artery stenosis and coronary heart diseases.

Poly(ADP-ribose) polymerase (PARP) 1 is a dominant member of the PARP family, accounting for approximately 90% of cellular PARP activity^4^. When cells encounter extrinsic or intrinsic stress signals, PARP1 can be activated immediately^5^. Upon activation, PARP1 poly(ADP-ribosyl)ates diverse acceptor proteins, exerting regulatory functions on multiple cellular processes, including DNA damage repair, transcriptional regulation, chromatin remodeling and mitotic apparatus regulation^4,5^. Transient and mild activation of PARP1 is commonly an important defense mechanism against cellular damage, whereas moderate, severe or sustained activation of PARP1 usually exacerbates the damage, even leading to cell death (apoptosis, necrosis and parthanatos)^4^. In the vasculature, activation of PARP1 has been implicated in the pathogenesis of inflammation and leukocyte infiltration, endothelial dysfunction, vascular cell apoptosis and necrosis^6–10^. Our group recently reported that PARP1 modulates the osteochondrocytic phenotypic change of VMSCs and upregulates the expression of mineralization-regulating proteins, thus contributing to vascular calcification^11^. However, the role of PARP1 in controlling the contractile status and mediating the VSMC synthetic phenotype switch remains largely unknown.

In this study, a robust activation of PARP1 was observed in VSMCs of injured arteries and atherosclerotic plaques in human and rodents. Gain- and loss-of-function studies revealed a pivotal role for PARP1 in the regulation of VSMC phenotype in vivo and in vitro. Upon activation, PARP1 poly(ADP-ribosyl)ated myocardin and myocardin-associated serum response factor (SRF), suppressed myocardin–SRF complex transactivation and contractile protein expression. Furthermore, poly(ADP-ribosyl)ation of myocardin and c-Jun abolished myocardin-induced suppression of c-Jun, enhancing VSMC proliferation and migration.

## Materials and methods

### Animal models

All experimental procedures were approved by the Animal Care Committee of Tongji Medical College, Huazhong University of Science and Technology (HUST). Male Sprague-Dawley rats (200–250 g, obtained from Tongji Medical College, HUST) were anesthetized by intraperitoneal administration of chloral hydrate (10%, 300 mg/kg). Balloon-catheter injury was performed in the left common carotid artery with a 2.0-French catheter as previously reported^12–14^. After balloon injury, 100 μl of viral solution containing indicated adenovirus at a titer of 3 × 10^9^ plaque-forming units was instilled into the isolated carotid segment and allowed to dwell for 20 min. After removal of the viral solution, the external carotid artery was ligated, and the blood flow to the common and internal carotid artery was restored. The left common carotid artery of sham operation controls (SOCs) was isolated without inducing injury. At indicated days (7, 14 and 21 days) after injury, rat carotid arteries were excised immediately and then used for the following experiments. For *N*-(6-oxo-5,6-dihydrophenanthoursidin-2-yl)-2-(*N*, *N*-dimethylamino) acetamide (PJ34, 10 mg/kg/day) treatment, rats were randomly grouped after operation and the PJ34 group rats received an intraperitoneal injection of PJ34 once a day. Male 8-week-old PARP1-knockout (PARP1^−/−^, 129S-Parp1tm1Zqw/J, PKO) mice and their wild-type (WT) 129Sv littermates were obtained from the Jackson Laboratory. After anesthetization, the left common artery of PKO or WT mice was exposed and then ligated. The ligation-injured segments were collected 2 or 4 weeks later. Contralateral noninjured carotid arteries were used as controls. Subsequent morphometric analyses were performed.

### Histological analyses and immunofluorescence staining

Rats or mice carotid arteries were fixed in 4% paraformaldehyde, embedded in paraffin. Tissues were cut into sequential slices for 5 μm and subjected to hematoxylin and eosin (H&E) staining. For immunofluorescence staining experiments, after high-pressure antigen retrieval process, the arterial sections were blocked in phosphate-buffered saline (PBS) with 10% goat serum for 1 h and incubated overnight with indicated primary antibodies and then washed with PBST and incubated with the appropriate secondary antibodies for 1 h. The nucleus was marked with 4′,6-diamidino-2-phenylindole (DAPI). The images were obtained using a fluorescence microscope. Antibodies for PAR (4338-MC-50, 4335-MC-100, 1:100) were from Trevigen. Antibodies for α-SMA (ab5694, 1:100) were from Abcam.

### Patient artery sample

All procedures involving human samples complied with the principles outlined in the Declaration of Helsinki and were approved by the institutional review board of Union Hospital, Tongji Medical College, HUST. Samples of human coronary artery after stenting (*n* = 4) were collected from patients with coronary artery diseases undergoing heart transplants who had ever received percutaneous coronary intervention. Atherosclerotic coronaries (*n* = 8) were collected from patients with coronary artery diseases for heart transplantation. Transplant renal artery stenosis samples (*n* = 4) were kindly provided by Tongji Hospital. The control internal mammary artery was obtained from patient who underwent coronary artery bypass.

### Whole-cell and nuclear extract

Tissues or cultured cells were homogenized in RIPA lysis buffer (50 mM Tris, 150 mM NaCl, 1% NP-40, 0.25% sodium deoxycholate and 1 mM EDTA, at a pH of 7.4) as whole-cell extracts. The buffer was supplemented with complete protease inhibitor (Roche) just before use. Nuclear extracts were prepared using hypotonic buffer (10 mM Tris, pH 7.4, 10 mM KCl, 1.5 mM MgCl_2_ and 1 mM EDTA). The cell or tissue suspension was passed through a 25 G needle on ice until no intact cells remained. The suspension was centrifuged at 500*g* for 10 min, and the pellet was dispersed in 500 µl ice-cold hypotonic buffer, then passed through a 25 G needle on ice again. The suspension was left on ice for an additional 5 min, then centrifuged at 500*g* for 10 min. The supernatant was discarded, and the pellet was washed with 1 ml of ice-cold hypotonic buffer. After centrifugation at 500*g* for another 10 min, the pellet was collected as the nuclear fraction. The nuclear fraction was then lysed using RIPA lysis buffer.

### Immunoblot assays

Proteins were determined using the bicinchoninic acid protein assay kit (Thermo Scientific, 23225). After denaturation and SDS–PAGE electrophoresis, separated proteins were transferred to polyvinylidene fluoride membranes (Millipore), which were incubated with 5% nonfat milk in TBST (50 mM Tris–HCl, pH 7.6, 150 mM NaCl and 0.2% Tween 20) and immunoblotted. Chemiluminescence signals were detected by the Image Lab software (Bio-Rad).

Antibodies for PAR (4338-MC-50, 4335-MC-100, 1:1,000) and PARP1 (AF-600-NA, 1:1,000) were from Trevigen. Antibodies for PARP1 (ab191217, 1:1,000) were from Abcam. Antibodies for FLAG (14793, 1:1,000), His (12698, 1:1,000), HA (3724, 1:1,000), SRF (5147, 1:1,000), c-Jun (9165, 1:1,000) and NF-κB p65 (8242, 1:1,000) were from Cell Signaling Technology. Antibodies for myocardin (sc-33766, 1:200), CEBPα (sc-365318, 1:1,000), MYB (sc-74512, 1:500) and SP1 (sc-17824, 1:1,000) were from Santa Cruz.

### Co-immunoprecipitation (Co-IP)

In brief, 300 µg of protein extracts were incubated with the indicated antibodies against PARP1, PAR, myocardin (Sigma-Aldrich, SAB4200539), SRF (Cell Signaling Technology, 5147), FLAG (Cell Signaling Technology, 14793), His (Cell Signaling Technology, 12698), HA (Cell Signaling Technology, 3724), c-Jun (Cell Signaling Technology, 9165), p65 (Cell Signaling Technology, 8242) or unspecific IgG at 4 °C overnight, and protein-A/G agarose was added for another 2–3 h at 4 °C. The immunoprecipitates were pelleted by centrifugation at 5,000 rpm for 4 min and washed three times with RIPA lysis buffer. The pellets were suspended in SDS gel loading buffer and subjected to western blot assays.

### Quantitative real-time PCR assay

Total RNA was isolated using TRIzol reagent (Invitrogen). For mRNA quantification, total RNA was reverse-transcribed by use of the use of Moloney Murine Leukemia Virus (MMLV) reverse transcriptase and oligo-(dT) primer or the PrimeScript RT Master Mix, and detected with SYBR Green PCR mix (Takara). The relative level of mRNA was calculated by the ∆∆Cq method with GAPDH as an internal control. For miRNA quantification, qRT–PCR was carried out using Bulge-LoopTM miRNA qRT-PCR Primer (RiboBio) following the manufacturer’s protocols. GAPDH was detected as the internal control. All the primer sequences used in this study are listed in Supplementary Table 1.

### Cell cultures

Primary mice and rat VSMCs (rVSMCs) were isolated by enzymatic digestion of thoracic aortic media from male C57BL/6J mice and Sprague-Dawley rats. Cells were maintained in Dulbecco’s modified Eagle medium (DMEM) with 20% fetal bovine serum. VSMCs at passages 3–7 were used for the experiments. T/G HA-VSMC, A7r5, HEK293 and HEK293T cells were purchased from the American Type Culture Collection and maintained in DMEM with 10% fetal bovine serum.

### Cell proliferation

VSMC proliferation in vitro was determined by 5-ethynyl-2′-deoxyuridine (EdU) incorporation assay using Cell-Light EdU DNA Cell Proliferation Kit (RiBoBio) or cell count. VSMCs were plated and quiesced in 0.2% fetal calf serum (FCS) media for 24 h before stimulation. The cells were incubated with or without platelet-derived growth factor BB (PDGF-BB, 20 ng/ml) for 12, 24 and 48 h. Cell counting was performed using a TC20 automated cell counter (Bio-Rad). During the last 2 h, EdU was added. Cell proliferation was quantified with EdU incorporation according to the manufacturer’s protocol.

### Transwell assay

VSMCs were added to the top wells of transwell-modified Boyden chambers of a 24-well transwell dish and then exposed to medium with or without PDGF-BB (20 ng/ml) that was added to the lower chamber. Six hours later, the cells that migrated to the bottom of membranes were stained with 0.1% crystal violet, photographed and counted.

### Wound healing assay

VSMCs were seeded onto six-well plates and then infected with indicated adenovirus for 24 h. A scratch wound was made with a 200-μl sterile pipette tip. The wounded VSMCs were washed twice with PBS to remove cell debris and further cultured in fresh DMEM containing PDGF-BB or not for indicated time. The cells were then observed and photographed. The wound area was calculated using ImageJ software.

### LC–MS/MS analysis of PARP1-associating proteins

For liquid chromatography–tandem mass spectrometry (LC–MS/MS) analysis of PARP1-associating proteins, VSMCs were transfected with the Flag-PARP1 vector or an empty vector for 48 h. Nuclear extracts were mixed with anti-FLAG M2 affinity gels (Sigma-Aldrich) and rotated overnight at 4 °C. The beads were washed with lysis buffer, and immunoprecipitated proteins were subjected to western blot and silver staining. The bands were then subjected to LC–MS/MS analysis. Data were analyzed with Protein Pilot software (AB SCIEX).

### Plasmid construction

The coding regions for human PARP1, myocardin and SRF were amplified by PCR and subcloned into the highly efficient mammalian expression plasmids p3×FLAG-CMV9, pcDNA3.1-His and pcDNA3.1-HA. Expression plasmids for PARP1 derivatives, PARP1△auto (with deletion of amino acids (a.a.) 477–524), were constructed using a PCR-mediated method and subcloned into the plasmid p3×FLAG-CMV9. Glutathione *S*-transferase (GST)-tagged PARP1 fragments A (a.a. 1–107), B (a.a. 108–214), C (a.a. 215–372), D (a.a. 373–476), E (a.a. 477–524), F (a.a. 525–656) and G (a.a. 657–1,014) and GST-tagged myocardin fragments A. (a.a. 1–131), B (a.a. 132–370), C (a.a. 371–561), D (a.a. 562–670), E (a.a. 671–839) and F (a.a. 840–987) were subcloned into the pGEX-4T-1 (Amersham Biosciences) vectors.

### GST pulldown

After isopropyl β-D-1-thiogalactopyranoside induction, 10 ml of *Escherichia coli* was collected, and the purified GST fusion protein was immobilized on glutathione-Sepharose 4B beads (GE Healthcare, Bio-Sciences AB). The GST–PARP1 and GST–myocardin beads were incubated with lysates of His-myocardin or Flag-PARP1-transfected 293T cells for 4 h at 4 °C. A GST tag was used as the negative control under the same conditions. The samples were washed four times and then analyzed by western blotting.

### Generation of recombinant adenovirus

The adenovirus kit, AdMax (Microbix), was used to generate adenovirus-based constructs according to the manufacturer’s recommendations. In brief, the recombinant shuttle plasmids were co-transfected with the genomic plasmid into HEK293 cells to produce the recombinant viral particles, and viral titers were enriched by two rounds of infection in HEK293 cells. To generate adenovirus encoding full-length human PARP1 (PubMed no. NM_001618.3), Flag-PARP1 cDNA fragments were transferred from pcDNA3-based vectors to the shuttle plasmid pDC316. The CMV-Null adenovirus was used as the negative control (Ad-Null).

To generate optional adenovirus-expressing short hairpin RNA (shRNA) against PARP1, myocardin, p65 and c-Jun, three lines of corresponding adenovirus were designed and constructed, then used to infect VSMCs to assess knockdown efficiency. The most efficient one was applied in the following experiments. The optional shRNA with the target sequence is listed in Supplementary Table 2. The negative control adenovirus was designed to express nontargeting ‘universal control’ shRNA (Scr shRNA).

### Luciferase assay

The promoter region of *SM22α*, *CyclinD1* and *MMP9* genes was amplified by PCR and subcloned into pGL3-Basic (Promega). The 6×CArG-Luc and its mutant constructs were designed with site-directed mutation in the CArG boxes (CC(A/T)6GG (WT) to AA(A/T)6GG or CC(A/T)6AA (mutant)).The 6×AP1-Luc and its mutant plasmids constructs were designed with site-directed mutation in the AP1-binding site (TGACTCA (WT) to GAACTAG (mutant)).These constructs were introduced into A7r5 or VSMC cells along with pSV-TK (Promega), which was used to normalize the transfection efficiency. Forty-eight hours after transfection, luciferase activities were measured using the dual-Luciferase Assay System (Promega).

### In vitro poly(ADP-ribosyl)ation assay

Nuclear extracts were incubated with different concentration of NAD^+^ in poly(ADP-ribosyl)ation assay buffer (50 mM Tris–HCl (pH 8.0), 10 mM MgCl_2_, 1 mM dithiothreitol and 3 ng/ml activated DNA) for 30 min at 37 °C. Poly(ADP-ribosyl)ated nuclear extracts were then subjected to co-IP or electrophoretic mobility shift assay (EMSA)^15,16^.

### EMSA assay

DNA–protein interactions were detected using a LightShift chemiluminescent EMSA kit (Thermo Scientific, 20148) according to the manufacturer’s protocol. The sequences of CArG-binding consensus oligonucleotides were as follows: 5′-GAGGTCCCTATATGGTTGT G-3′ (CArG). The oligonucleotides were labeled with biotin at their 5′ ends^17^. In brief, binding reactions with reaction mixtures containing 20 μg of nuclear extracts and 50 fM oligonucleotide were performed for 30 min in binding buffer (2.5% glycerol, 0.05% Nonidet P-40, 50 mM KCl, 5 mM MgCl_2_, 1 mM EDTA, 10 mM Tris (pH 7.6) and 50 ng poly(dI-dC)). The unlabeled probe, at a concentration 200-fold that of the labeled probe, was used as a competitor. Protein–DNA complexes were subjected to 6% native polyacrylamide gel electrophoresis and thereafter were transferred to nylon membranes (Pierce), which were immediately cross-linked on an ultraviolet transilluminator. Bands were then detected by the chemiluminescent method according to the manufacturer’s protocol.

### Chromatin immunoprecipitation (ChIP) and repeated-ChIP (re-ChIP) assay

For the ChIP assay, rVSMCs were treated according to the procedure. After 48 h, cells were cross-linked with 1% formaldehyde for 10 min, stopped by adding glycine to a final concentration of 125 mM, then collected and sonicated to generate DNA fragments of 0.25–1 kb. Lysates were centrifuged, and an aliquot of supernatant was saved as input DNA. Supernatants were then immunoprecipitated with indicated antibodies or an IgG as a negative control. Finally, DNA was purified using the QIAquick PCR purification kit. Purified DNA was analyzed by real-time PCR with specific primers for SM22α and CyclinD1 promoter. In the re-ChIP assay, chromatin was first immunoprecipitated with an anti-SRF or anti-c-Jun antibody, then eluted with 100 μl of elution buffer with 10 mM dithiothreitol at 37 °C for 30 min, diluted with dilution buffer and finally re-immunoprecipitated with IgG or an antibody against PARP1, PAR or myocardin.

### Gene expression profiling by RNA-seq analysis

Total RNAs of rVSMCs with two biological replicates were extracted. After the construction of the RNA sequencing (RNA-seq) library, the libraries were sequenced using BGI500 for 50-bp paired-end sequencing. For RNA-seq data analysis, we used Fastqc for raw data quality control and Cutadapt to remove low-quality reads and adaptor contaminations. After data filtering, Tophat2 was used for data mapping to rat genome rn6. We accepted only two mismatches for each read. Cufflinks was used for transcript assemble and differential expression gene analysis with replicates (false discovery rate (FDR) <0.05%). Gene Ontology (GO) analysis was performed using DAVID. Gene set collections from the Molecular Signatures Database (MSigDB) 3.0 were used for the analysis of the SRF, c-Jun and p65 transcriptomes. The sequences presented in this Article have been submitted to the Gene Expression Omnibus under accession number GSE103995.

### Study approval

All procedures involving human samples complied with the principles outlined in the Declaration of Helsinki and were approved by the institutional review board of Union Hospital, Tongji Medical College, HUST. All participants signed informed consent before entering this study. Experimental animal protocols were approved by the Institutional Animal Care and Use Committee of Tongji Medical College, HUST.

### Statistical analysis

Values are shown as the means ± s.d. of at least three independent experiments. Homogeneity of the variance was assessed by the *F* test (two groups) or Brown–Forsythe test (three or more groups). The statistical significance of differences between two groups was analyzed by Student’s *t*-tests. For comparing more than two means, one-way analysis of variance with the Bonferroni post-hoc analysis was used. Values of *P* < 0.05 were considered statistically significant. All statistical analyses were performed using SPSS software (version 22.0, SPSS).

## Results

### PARP1 is activated in VSMCs of artery stenosis and atherosclerotic plaques

**Fig. 1:**
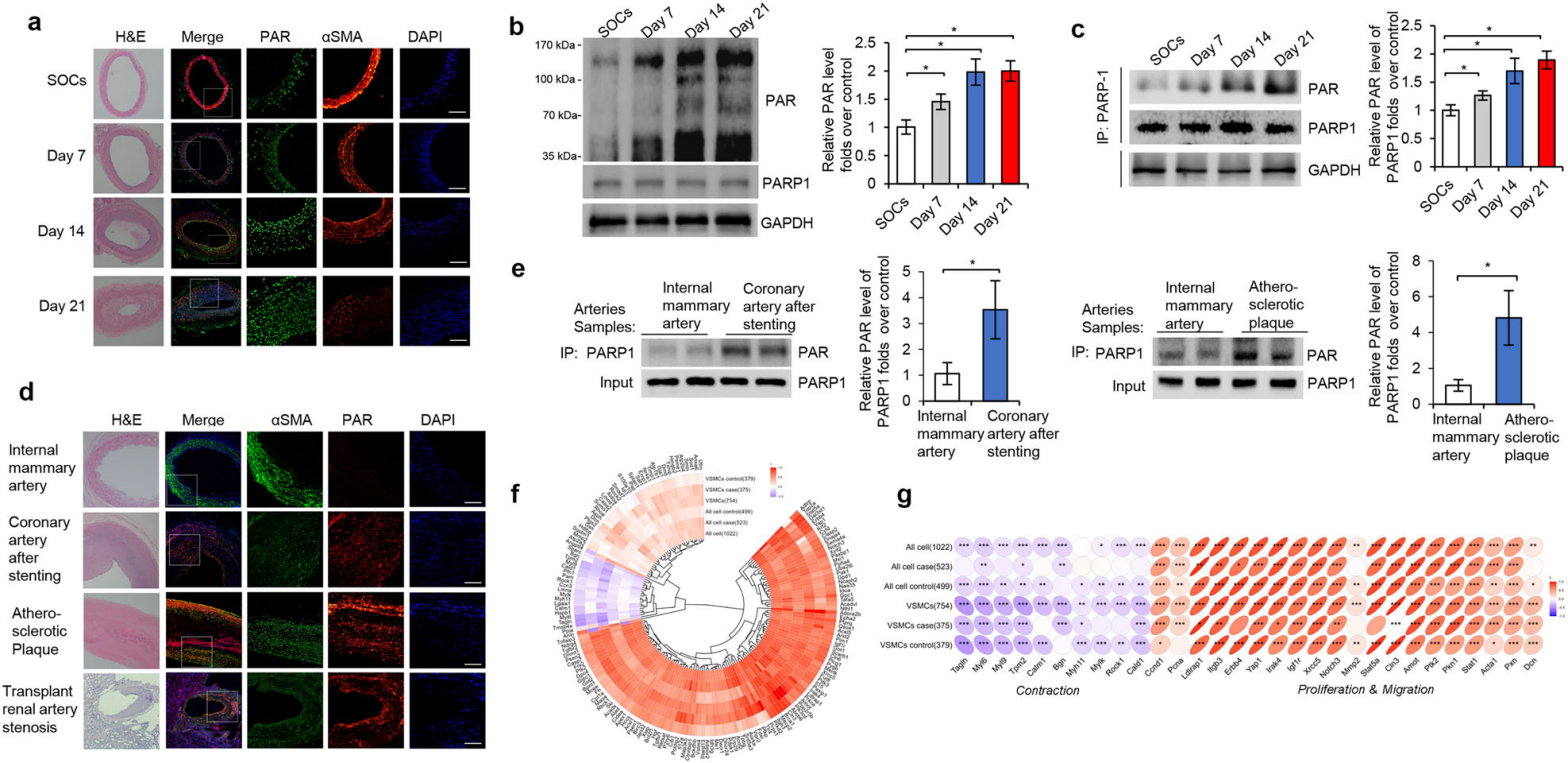
PARP1 is activated in VSMCs undergoing phenotype switch. **a**, Double immunofluorescence staining for poly(ADP-ribose) polymer (PAR, green) and VSMC marker αSMA (red) in SOCs and balloon-injured rat carotid arteries after injury (*n* = 5–8 for each time point). Nuclei were stained with DAPI (blue). **b**, Immunoblot assay of poly(ADP-ribosyl)ated proteins in lysates of SOCs and balloon-injured arteries at various time points after injury. **c**, PARP1 was immunoprecipitated from lysates of SOCs and balloon-injured arteries, followed by immunoblot with anti-PAR antibody (top); PARP1 expression in the lysates was determined using immunoblot (bottom) (*n* = 5). **d**, Double immunofluorescence staining for PAR (red) and αSMA (green) in human samples of normal internal mammary artery (*n* = 6), coronary artery after stenting (*n* = 4), coronary atherosclerotic plaque (*n* = 8) and transplant renal artery stenosis (*n* = 4). **e**, PARP1 was immunoprecipitated from lysates of human internal mammary artery and coronary artery after stenting (*n* = 4, left) or atherosclerotic plaque (*n* = 6, right), followed by immunoblot with anti-PAR antibody. **f**, **g**, Reanalysis of previously reported single-cell RNA-seq dataset generated from balloon-injured carotid arteries of rats (GSE174098) showing that a majority of genes involved in cell proliferation and migration were positively correlated with PARP1 expression, and those pathways related to VSMC contraction were negatively associated with mRNA level of PARP1. **f** shows the Circos heatmap of genes associated with PARP1, and **g** provides a detailed view of the genes involved in cell contraction, proliferation, and migration. Scale bar, 50 μm. Throughout, data are presented as mean ± s.d. *P < 0.05.

To explore whether PARP1 was involved in the VSMC phenotype switch, we first examined PARP activity in VSMCs of rat balloon-injured carotid arteries. Compared with SOCs, ballon-injured arteries manifested a time-dependent upregulation of poly(ADP-ribose) polymer (PAR) and VSMC marker (αSMA)^1^ double-positive cells in the neointima and media (Fig. 1a), indicating that PARP activity was increased in VSMCs after injury. In line with this, western blotting revealed a time-dependent increase in total PAR levels in the neointima and media (mainly VSMCs) (Fig. 1b). Together, these results indicated that PARP activity was increased in VSMCs undergoing phenotype switch in vivo. The PARP family of enzymes consists of 17 members^5,18^. Immunoprecipitation (IP) assay showed that balloon-injured arteries versus SOCs exhibited a time-dependent increase in poly(ADP-ribosy)lation of PARP1 (reflecting PARP1 activity) in the neointima and media (Fig. 1c), suggesting that PARP1 was activated in VSMCs.

We next examined whether PARP1 was activated in VSMCs of human samples. Compared with healthy mammary arteries, samples of coronary arteries after stenting (*n* = 4), coronary atherosclerotic plaques (*n* = 6) and transplant renal artery stenosis (*n* = 4) displayed upregulated PAR/αSMA double-positive cells in the lesions (Fig. 1d). Moreover, IP revealed that poly(ADP-ribosy)lation levels of PARP1 increased markedly in these lesions (Fig. 1e). Therefore, PARP1 was activated in VSMCs of human artery stenosis and atherosclerotic plaques.

### PARP1 contributed critically to VSMC phenotype switch in vivo

SMC phenotype modulation is critically important for the resolution of vascular injury, such as restenosis following angioplasty. We analyzed the single-cell RNA-seq dataset generated from balloon-injured carotid arteries of rats (GSE174098). Interestingly, our data revealed that a majority of genes involved in cell proliferation and migration were positively correlated with PARP1 expression across tissue groups, and those pathways related to VSMC contraction were negatively associated with mRNA level of PARP1 (Fig. 1f, g). Single-cell RNA-seq data suggest that PARP1 may act as a potential upstream driver of smooth muscle cell modulation. To further explore whether PARP1 was involved in VSMC phenotype switch after balloon injury, qRT–PCR revealed that PJ34 (9*N*-(6-oxo-5,6-dihydrophenanthridin-2-yl)-*N*,*N*-dimethylacetamide), a PARP inhibitor, upregulated contractile genes (*αSMA*, *Sm22α*, *Cnn1* and *Myh11*) but downregulated proliferative gene (*CyclinD1*, *Pcna*, *CyclinA2* and *Cdk6*) and migratory gene (*Mmp9*, *Pxn*, *Ptk2*, *Itgb3, Tnc* and *Mmp2*) expression (Supplementary Fig. 1a). Consistently, the neointima/media ratio was reduced after PJ34 treatment (Supplementary Fig. 1b). Taken together, pharmacological inhibition of PARP1 suppressed the VSMC phenotype switch.

**Fig. 2:**
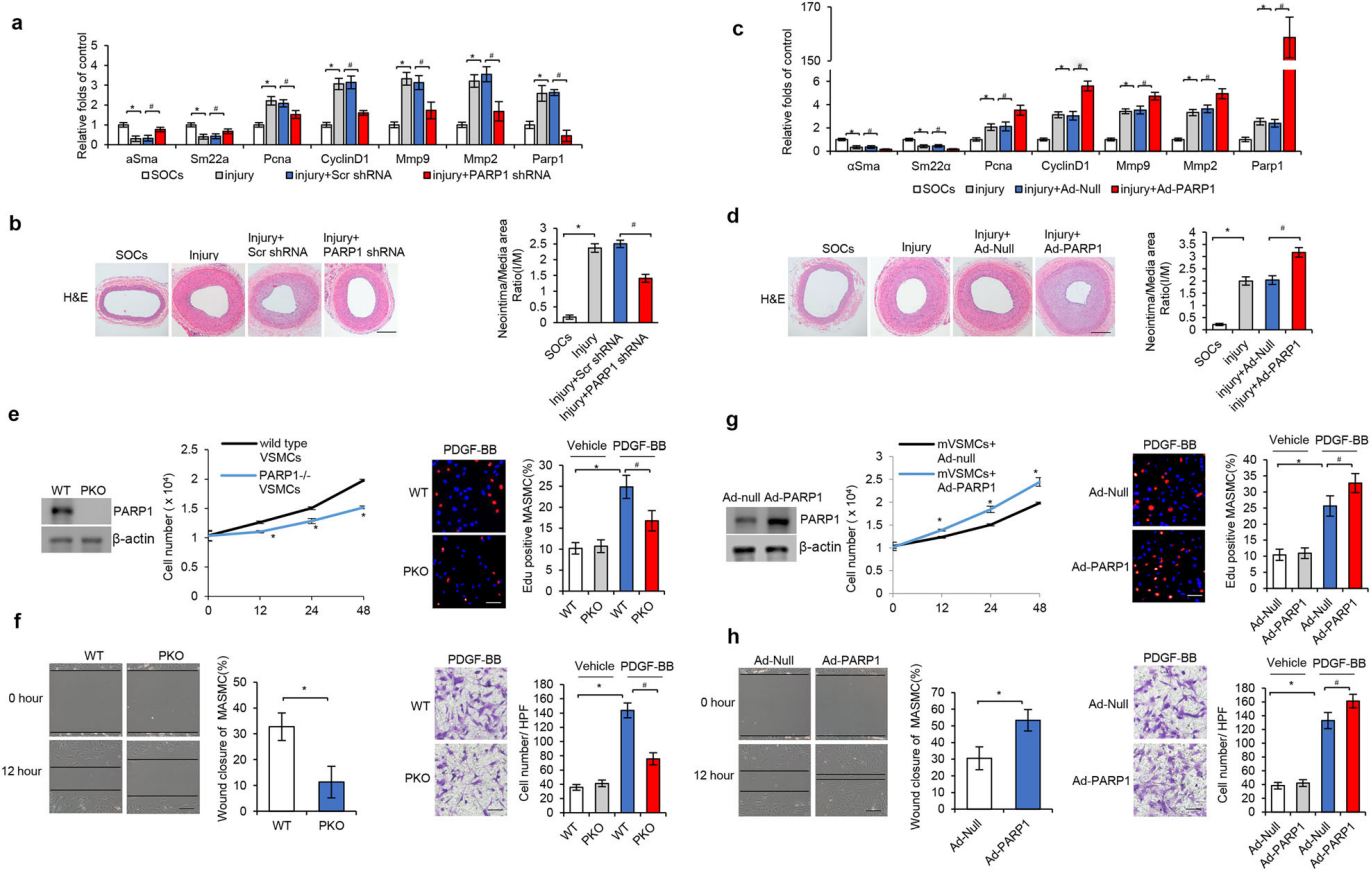
PARP1 contributes to VSMC phenotype switch in vivo and in vitro. **a**, **b**, Balloon-injured rat carotid arteries were in-site infected with adenoviruses encoding PARP1 shRNA (Ad-PARP1 shRNA) or negative control (Ad-Scr shRNA) immediately after operation, and collected 14 days later (*n* = 5-8). The mRNA levels of *αSMA*, *Sm22α*, *CyclinD1*, *Pcna*, *Mmp2*, *Mmp9* and *Parp1* (**a**), and H&E staining for neointima and the neointima/media ratio (**b**). **c**, **d**, Balloon-injured rat carotid arteries were in-site infected with adenoviruses encoding human PARP1 (Ad-PARP1) or empty vector (Ad-Null) immediately after operation, and collected 10 days later (*n* = 5-8). The mRNA levels of *αSMA*, *Sm22α*, *CyclinD1*, *Pcna*, *Mmp2, Mmp9* and *Parp1* (**c**), and H&E staining for neointima the neointima/media ratio (**d**). Scale bar, 50 μm**. e**, **f**, Primary VSMCs from WT and PKO mouse (mVSMCs) were cultured and treated with PDGF-BB (20 ng/μl): cell proliferation was determined using the cell counting assay (at 0, 12, 24 and 48 h, left) and EdU staining at 48 h (right) (*n* = 4) (**e**); representative images of migrated mVSMCs, 12 h after the initiation of wound healing assay (left) and 6 h after the initiation of transwell migration assay (right) (*n* = 4) (**f**). **g**, **h**, Ad-Null- or Ad-PARP1-infected mVSMCs were treated with PDGF-BB (20 ng/μl) (*n* = 4): mVSMC proliferation was determined using cell counting assay (left) and EdU staining (right) (*n* = 4) (**g**); mVSMC migration was determined using wound healing assay (left) and transwell migration assay (right) (*n* = 4) (**h**). Scale bar, 20 μm. Throughout, data are presented as mean ± s.d. For **a**–**d**, *P < 0.05 versus SOCs; ^#^*P* < 0.05 versus injury + Scr shRNA or injury + Ad-Null; for **e**–**h**, *P < 0.05 versus WT or Ad-Null; ^#^*P* < 0.05 versus PDGF-BB + WT or PDGF-BB + Ad-Null.

In line with this, infection of balloon-injured arteries with adenovirus encoding shRNA against PARP1 (PARP1 shRNA) versus scramble shRNA reserved the expressional alteration of genes involved in contraction, suppressed the genes in migration and proliferation (Fig. 2a) and attenuated the neointima/media ratio (day 14) (Fig. 2b). These results indicated that deficiency in PARP1 suppressed the VSMC phenotype switch.

In experiments of PARP1 gain of function, infection with adenovirus encoding human PARP1 gene (Ad-PARP1) versus empty vector (Ad-Null) suppressed contractile gene but increased proliferative and migratory gene expression, and increased neointima/media ratio of balloon-injured arteries (day 10) (Fig. 2c, d), indicating that gain of PARP1 promoted the VSMC phenotype switch.

### PARP1 exerted important functions in PDGF-BB-induced proliferation and migration of VSMCs

PDGF-BB is an important mitogen promoting VSMC proliferation and migration. We next determined whether PARP1 contributed to the PDGF-BB-induced VSMC phenotype switch. After PDGF-BB stimulation, PKO-m in VMSCs is mouse versus wt in wt-mVSMCs is Wild type displayed a decreased cell number and less EdU incorporation (Fig. 2e), indicating that deficiency in PARP1 suppressed VSMC proliferation. Similarly, PKO-m in VMSCs is mouse versus wt in wt-mVSMCs is Wild type displayed a reduced wound closure and transwell migration after PDGF-BB treatment (Fig. 2f), indicating that deficiency in PARP1 suppressed VSMC migration. In experiments of PARP1 gain of function, infection with Ad-PARP1 versus Ad-Null increased cell numbers, EdU incorporation, wound closure and transwell migration of wt-mVSMCs under PDGF-BB treatment (Fig. 2g, h), indicating that gain of PARP1 promoted VSMC proliferation and migration.

### Inhibition of PARP1 effectively reversed PDGF-BB-induced transcriptome alteration of VSMCs

**Fig. 3:**
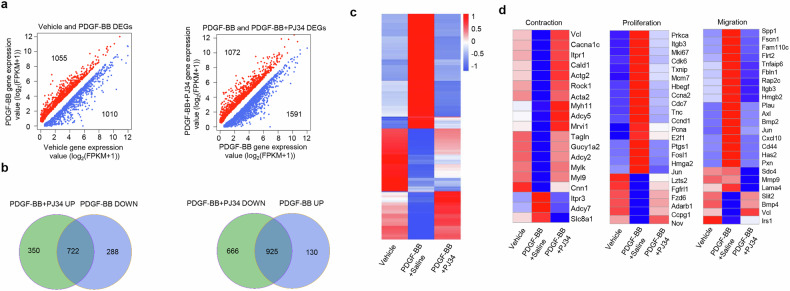
PARP1 contributes to PDGF-BB-induced transcriptome alteration of VSMCs. Primary rVSMCs were treated with vehicle, PDGF-BB or PDGF-BB + PJ34(10 μM) for 48 h, and the gene transcription profiles were analyzed using RNA-seq. **a**, Scatter plots of DEGs between PDGF-BB libraries and vehicle (left), and DEGs between PDGF-BB + PJ34 and PDGF-BB-treated libraries (right). Red dots represent related higher DEGs, and blue dots represent lower DEGs. **b**, A Venn diagram showing the intersection of genes downregulated by PDGF-BB treatment and upregulated by PJ34 (rd-DEGs, left), and the intersection of genes upregulated by PDGF-BB but downregulated in PDGF-BB + PJ34 libraries (ru-DEGs, right). **c**, A heat map of DEGs among vehicle, PDGF-BB and PDGF-BB + PJ34 groups. **d**, Genes related to VSMC contraction, proliferation and migration were separated and illustrated.

We then examined the effects of PARP1 inhibition on PDGF-BB-induced transcriptome alteration of rVSMCs. PDGF-BB treatment resulted in 2,065 differentially expressed genes (DEGs, expected FDR <0.05), among which 1,010 genes were downregulated and 1,055 genes were upregulated (Fig. 3a). Simultaneous treatment with PDGF-BB plus PJ34 reversed the transcripts of 722 in 1,010 DEGs downregulated by PDGF-BB (rd-DEGs) and 925 in 1,055 DEGs upregulated by PDGF-BB (ru-DEGs) (Fig. 3b). The heat map shows that PJ34 treatment notably reversed PDGF-BB-induced transcriptome alteration (Fig. 3c).

GO analysis revealed that 722 rd-DEGs were enriched in the processes of vascular constriction, cell migration and proliferation (*P* < 0.01). Kyoto Encyclopedia of Genes and Genomes (KEGG) pathway analysis revealed a strong association between 722 rd-DEGs and pathway of vascular smooth muscle contraction (*P* < 0.01) (Supplementary Fig. 2a). As for 925 ru-DEGs, they were enriched in cell division, DNA replication, cell cycle, regulation of cell migration and proliferation (*P* < 0.01). KEGG pathway analysis revealed a significant association between 925 ru-DEGs and pathways of cell cycle and DNA replication (*P* < 0.01) (Supplementary Fig. 2b). In line with these results, the heat map shows that PJ34 reversed PDGF-BB-induced alteration of transcript profiles associated with VSMC contraction, proliferation and migration (Fig. 3d).

### PARP1 interacted with and poly(ADP-ribosyl)ated myocardin in the nucleus

**Fig. 4:**
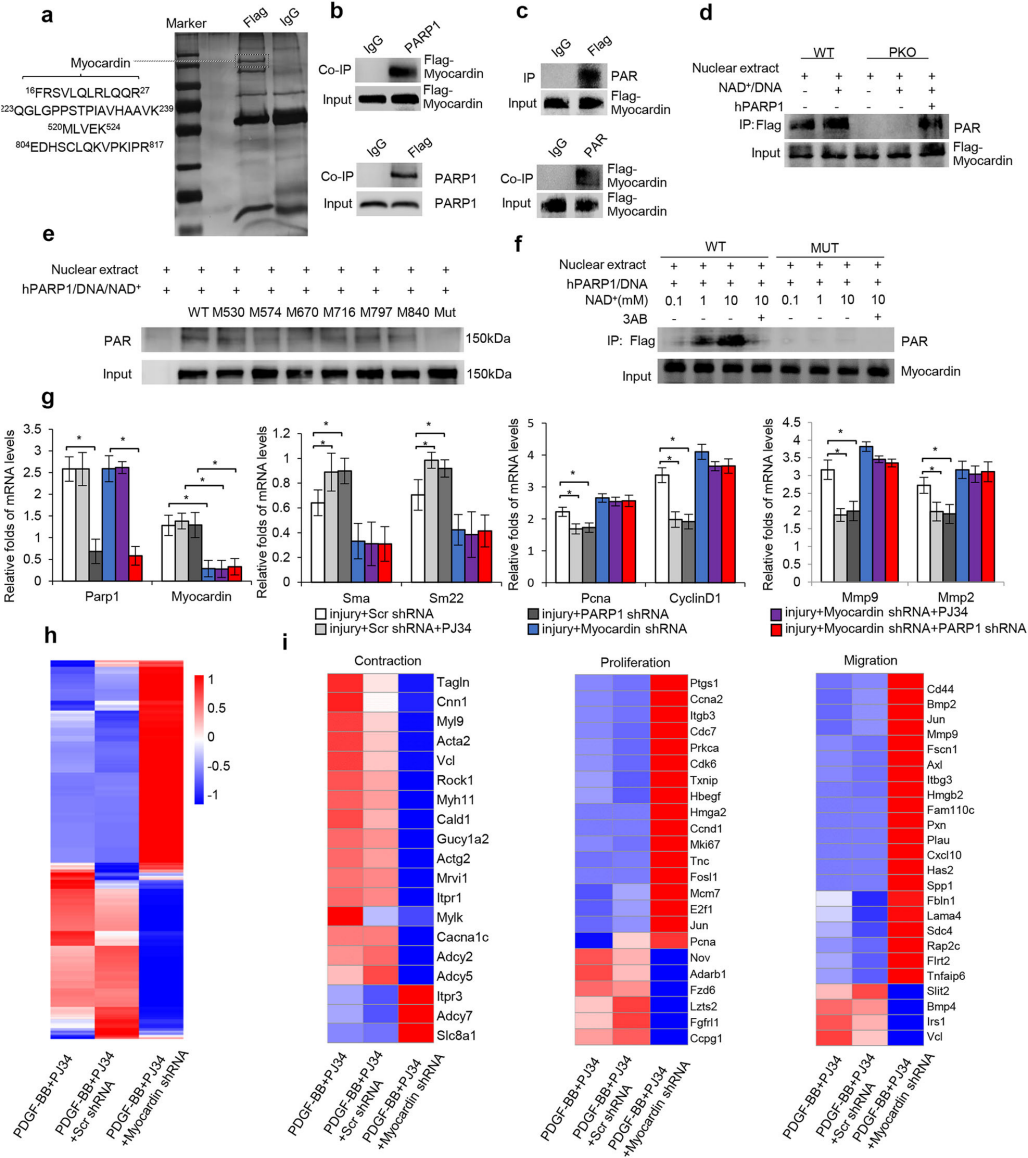
Myocardin mediates the effects of PARP1 inhibition on VSMC phenotype switch and transcriptome alteration. **a**, PARP1-containing protein complexes were immunoaffinity purified from T/G-HAVSMC nuclear extracts and then subjected to SDS–PAGE and analyzed by LC–MS/MS assay. Black letters indicate the peptides identified. **b**, Co-IP assay of PARP1-bound proteins and myocardin-bound proteins in nuclear extracts from T/G-HAVSMCs infected with Flag-myocardin adenovirus, followed by immunoblot with anti-Flag antibody (top) and with anti-PARP1 antibody (bottom) (*n* = 3). Nonspecific IgG served as a negative control. **c**, IP of myocardin and poly(ADP-ribosyl)ated proteins in nuclear extracts from T/G-HAVSMCs infected with Flag-myocardin adenovirus, followed by immunoblot with anti-PAR antibody (top) and with anti-Flag antibody (bottom) (*n* = 3). Nonspecific IgG served as a negative control. **d**, Poly(ADP-ribosyl)ation of myocardin immunoprecipitated from nuclear extracts of mVSMCs infected with Flag-myocardin adenovirus, which were preincubated with active DNA/NAD^+^ alone or together with recombinant human PARP1 protein (hPARP1), was determined using immunoblot. **e**, Poly(ADP-ribosyl)ation of myocardin immunoprecipitated from nuclear extracts of T/G-HAVSMCs transfected with different mutant constructs, which were preincubated with active DNA/NAD^+^ alone or together with recombinant human PARP1 protein (hPARP1), was determined using immunoblot. **f**, Poly(ADP-ribosyl)ation of myocardin in nuclear extracts from VSMCs transfected with WT or mutant myocardin plasmid, which were incubated with active DNA/NAD^+^ alone or together with hPARP1 or 3AB, was determined using immunoblot. **g**, Balloon-injured rat arteries were in-site infected with Ad-Scr shRNA, Ad-PARP1 shRNA or adenoviruses encoding myocardin shRNA (Ad-myocardin shRNA) immediately after injury and then were treated with PJ34 for 10 days (*n* = 5–8). mRNA levels of *Parp1*, *myocardin*, *αSMA*, *Sm22α*, *CyclinD1*, *Pcna, Mmp2* and *Mmp9* in injured arteries at 10 days. **h**, **i**, rVMSCs were infected with Ad-myocardin shRNA or Ad-Scr shRNA and then treated with PDGF-BB + PJ34 for 48 h. The gene transcription profiles were analyzed by RNA-seq: heat map of DEGs (**h**); genes related to VSMC contraction, proliferation and migration were separated (**i**). Throughout, data are presented as mean ± s.d. *P < 0.05 versus injury + Scr shRNA or injury + myocardin shRNA.

To identify proteins responsible for mediating the effects of PARP1, Flag-tagged PARP1 (Flag-hPARP1) was introduced into human aortic VSMC cell line T/G HA-VSMCs. After IP of nuclear extracts with anti-Flag antibody, infection with Flag-hPARP1 versus empty vector resulted in an additional band with molecular weight around 150 kDa in eluted proteins (Fig. 4a). After elimination of false positives presenting in empty vector controls, LC–MS/MS assay identified this protein as myocardin (Fig. 4a), a master regulator of contractile genes in VSMCs^19,20^. This finding was confirmed by co-IP assay, which showed that PARP1 was co-precipitated with Flag-myocardin in the nuclear extracts, and vice versa (Fig. 4b). Furthermore, GST pulldown assay localized the myocardin-binding site to the BRCT/auto-modification domain of PARP1 and the PARP1-binding site to the C-terminal transcription activation domain of myocardin (Supplementary Fig. 3a). In support of this, myocardin could not interact with PARP1 mutants lacking the BRCT/auto-modification domain (PARP1Δauto) (Supplementary Fig. 3b).

To determine whether myocardin could be poly(ADP-ribosyl)ated by PARP1, nuclear extracts from T/G HA-VSMCs infected with Flag-myocardin adenovirus were immunoprecipitated with anti-Flag antibody. Western blotting showed that myocardin was poly(ADP-ribosyl)ated (Fig. 4c). Consistently, myocardin was detected among nuclear proteins precipitated with anti-PAR antibody (Fig. 4c). Moreover, because poly(ADP-ribosyl)ated myocardin was not detected in PKO cells (Fig. 4d), we suspected that it might be poly(ADP-ribosyl)ated by PARP1. Indeed, incubation of nuclear extracts from PKO-mVSMCs with recombinant human PARP1 protein (hPARP1) led to poly(ADP-ribosyl)ation of myocardin (Fig. 4d). Taken together, myocardin was poly(ADP-ribosyl)ated by PARP1 in VSMCs.

Next, we determined the residue(s) of myocardin that can be poly(ADP-ribosyl)ated by PARP1. LC–MS/MS analysis was conducted in parallel on trypsin digests of purified, native myocardin after in vitro poly(ADP-ribosyl)ation by PARP1 and allowed a sequence coverage of 98%. Data interpretation of the corresponding MS/MS spectra unambiguously identified Glu530, Glu574, Glu670, Glu716, Asp797 and Asp840 sites in samples of poly(ADP-ribosyl)ated myocardin. Incubation of nuclear extracts from VSMCs with hPARP1 led to poly(ADP-ribosyl)ation of myocardin, while the poly(ADP-ribosyl)ation of myocardin was completely abrogated in cells transfected with all sites-directed mutant construct (myocardin D530A + D574A + D670A + D716A + E797A + E840A) (Fig. 4e, f). Together, these data indicate that Glu530, Glu574, Glu670, Glu716, Asp797 and Asp840 are required for poly(ADP-ribosyl)ation of myocardin.

### Depletion of myocardin reversed the PARP1 inhibition-mediated phenotype switch in VSMCs

Here, infection with adenovirus encoding shRNA against myocardin (Ad-myocardin shRNA) versus scramble shRNA abrogated PJ34- or shPARP1-induced expressional alteration of genes involved in VSMC contraction, proliferation and migration (Fig. 4g), as well as reduction in the neointima/media ratio of balloon-injured arteries (Supplementary Fig. 4a). In PDGF-BB-treated rVSMCs, infection with Ad-myocardin shRNA abolished PJ34- or shPARP1-induced suppression of EdU incorporation and wound closure (Supplementary Fig. 4b,c). Therefore, deletion of myocardin abrogated the effects of PARP inhibition on VSMC phenotype switch.

Next, the effects of myocardin deletion on transcriptome of VSMCs treated with both PJ34 and PDGF-BB were examined. Infection of rVSMCs with Ad-myocardin shRNA versus scramble shRNA resulted in 5,271 DEGs (FDR <0.05), among which 2,903 genes were upregulated and 2,368 genes were downregulated (Supplementary Fig. 5a). The heat map shows that Ad-myocardin shRNA infection resulted in a transcriptome pattern nearly opposite that of scramble controls (Fig. 4h). GO analysis showed that 5,271 DEGs were significantly enriched in cell (VSMC) migration and proliferation (*P* < 0.01). KEGG pathway analysis revealed a strong association between 5,271 DEGs and pathways of DNA replication, cell cycle and vascular smooth muscle contraction (*P* < 0.01) (Supplementary Fig. 5b). Specifically, heat maps showed that infection with Ad-myocardin shRNA resulted in opposite transcript patterns of genes involved in contraction, proliferation and migration to scramble controls (Fig. 4i), indicating a critical role for myocardin in PJ34-induced transcript alteration of these genes.

### Myocardin-mediated poly(ADP-ribosyl)ation of SRF by PARP1

**Fig. 5:**
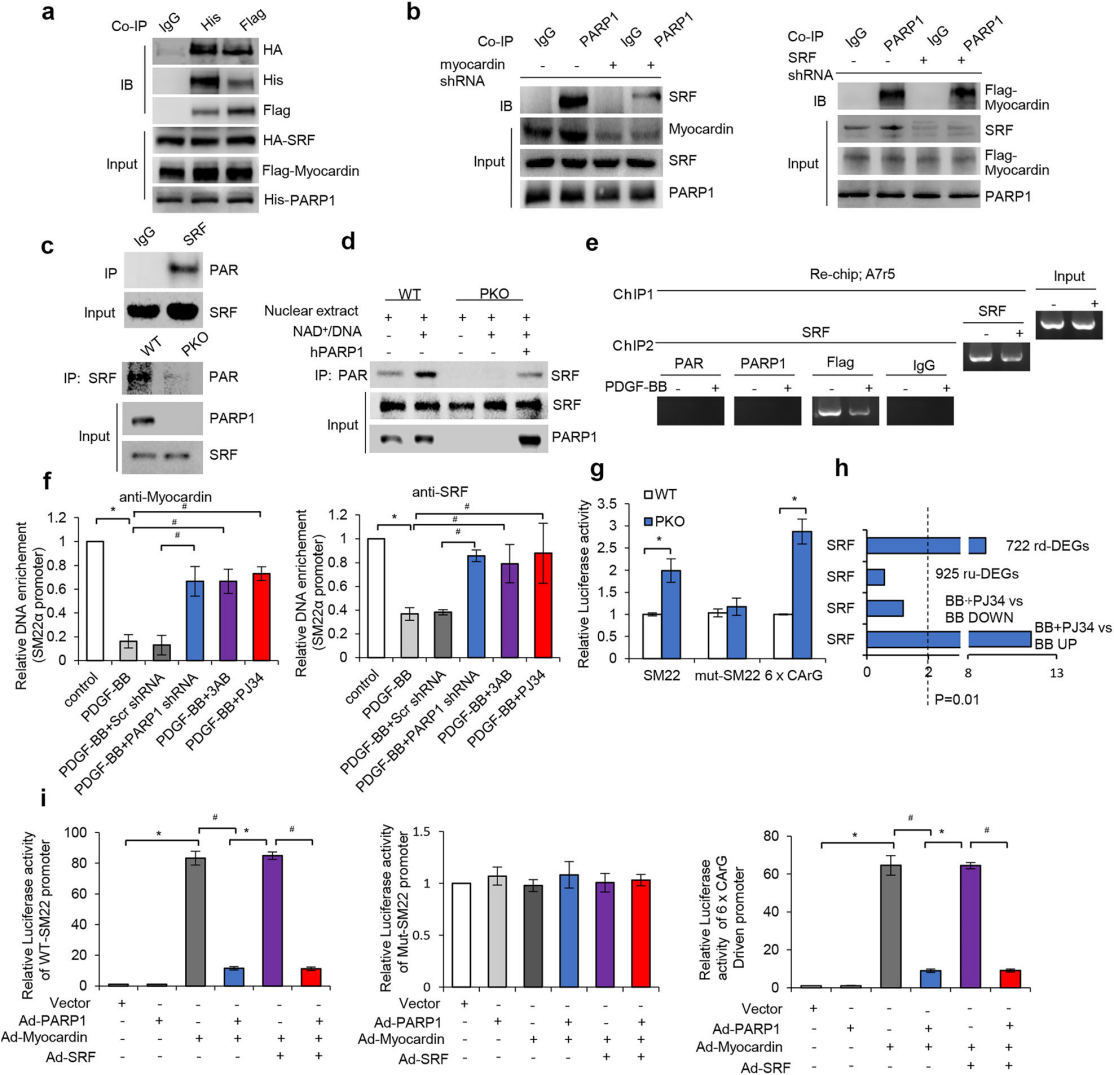
PARP1-mediated poly(ADP-ribosyl)ation of myocardin and SRF suppresses the complex transactivation and target transcription. **a**, The interaction among His-tagged PARP1, Flag-tagged myocardin and HA-tagged SRF in 293T cells was determined using Co-IP assay. **b**, A7r5 cells were infected with Ad-myocardin shRNA or Ad-SRF shRNA. PARP1-bound SRF and myocardin was detected using co-IP assays (*n* = 3). **c**, Poly(ADP-ribosyl)ation of SRF immune- precipitated from T/G-HAVSMC nuclear extracts (top) or aortic lysates of WT and PKO mouse (bottom) was determined using immunoblot. **d**, Poly(ADP-ribosyl)ation of SRF in nuclear extracts from WT-mVSMCs or PKO-mVSMCs, which were incubated with active DNA/NAD^+^ alone or together with hPARP1, was determined using immunoblot. **e**, A7r5 cells infected with Flag-myocardin adenovirus, and exposed to PDGF-BB treatment for 48 h. Chromatin was first immunoprecipitated with anti-SRF antibody, and then re-immunoprecipitated with anti-PAR, anti-PARP1, anti-Flag (myocardin) or anti-IgG antibody. **f**, ChIP assay using anti-myocardin (left) or anti-SRF (right) for amplification of rat *SM22α* promoters in rVSMCs treated with PARP inhibitors (3AB, PJ34) or infected with Ad-PARP1 shRNA in the presence of PDGF-BB for 48 h. **g**, Luciferase reporters driven by WT or CArG-mutant *SM22α* promoter (WT-*SM22α* and MUT-*SM22α*, respectively) or 6×CArG were transfected into PDGF-BB-treated WT- or PKO-mVSMCs, and luciferase activities were thereafter analyzed (*n* = 4). **h**, TF target gene sets were collected from the MSigDB C3 TFT motif gene sets, and multiple target gene sets of certain TF were combined to serve as an integrated targets source, including SRF_01, SRF_C, SRF_Q4, SRF_Q5_01 and SRF_Q6. Fisher’s exact test was used to detect TF targets overrepresentation in DEGs. **i**, A7r5 cells were preinfected with Ad-PARP1, Ad-myocardin and Ad-SRF as indicated, and the luciferase activities driven by WT-SM22α (left), MUT-SM22α (middle) or 6×CArG (right) were determined at 48 h after reporter transfection (*n* = 4). Throughout, data are presented as mean ± s.d. *P < 0.05 versus control or WT or Vector, ^#^*P* < 0.05 versus *P*DGF-BB or PDGF-BB + Scr shRNA or Ad-myocardin or Ad-myocardin + Ad-SRF.

Myocardin forms a higher-order complex with SRF to regulate transcription^20,21^. To determine whether SRF interacted with PARP1, HEK293T cells were simultaneously transfected with pcDNA3.1-His-myocardin plasmid, p3×FLAG-CMV9-PARP1 plasmid and pcDNA-3.1-HA-SRF plasmid, and thereafter, co-IP using anti-tag antibodies was used. As expected, PARP1, myocardin and SRF could coprecipitate with each other (Fig. 5a). Moreover, SRF deficiency failed to disrupt PARP1–myocardin interaction, but myocardin depletion impaired PARP1–SRF binding (Fig. 5b), suggesting that myocardin mediated the interaction between PARP1 and SRF.

In this study, poly(ADP-ribosyl)ated SRF was detected in the nuclear extracts from T/G HA-VSMCs or wt-mVSMCs, but not from PKO-mVSMCs, suggested that SRF might be poly(ADP-ribosyl)ated by PARP1(Fig. 5c). In support of this, incubation of nuclear extracts from PKO-mVSMCs with hPARP1 led to poly(ADP-ribosyl)ation of SRF (Fig. 5d), indicating that SRF was poly(ADP-ribosyl)ated by PARP1 in VSMCs.

### PARP1 suppressed myocardin–SRF transactivation through poly(ADP-ribosyl)ation of them

The myocardin–SRF complex binds to CArG box in contractile gene promoter to facilitate transcription^21,22^. Re-ChIP showed that poly(ADP-ribosy)lated proteins did not recruit to the SM22α gene promoter (a target of myocardin–RF) (Fig. 5e), indicating that poly(ADP-ribosyl)ation of myocardin–SRF suppressed the complex binding to target promoter. In line with this, ChIP–qPCR showed that infection with shPARP1 or treatment with PARP inhibitor (3AB or PJ34) increased recruitment of myocardin–SRF to *SM22α* promoters (Fig. 5f). Moreover, EMSA assay showed that incubation of nuclear extracts from T/G HA-VSMCs with NAD^+^/active DNA, which promoted endogenous myocardin and SRF poly(ADP-ribosyl)ation, suppressed binding of myocardin–SRF to the αSMA-CArG probe (Supplementary Fig. 6), indicating that poly(ADP-ribosyl)ation decreased the DNA binding capacity of the myocardin–SRF complex.

Luciferase dual-reporter assay showed that PKO-mVSMCs versus wt-mVSMCs displayed a much higher luciferase activity driven by WT *SM22α* promoter (Fig. 5g). Because luciferase activity driven by the CArG box-mutated (MUT) *SM22α* promoter was not affected, this result indicated that deficiency in PARP1 enhanced myocardin–SRF-dependent promoter activation. In line with this, PKO-mVSMCs versus wt-mVSMCs displayed a threefold higher luciferase activity driven by six tandem repeats of CArG box (6×CArG). Together, deficiency in PARP1 promoted myocardin–SRF transactivation in PDGF-BB-treated cells. Consistently, enrichment analysis showed that simultaneous treatment with PDGF-BB and PJ34 resulted in a significant overrepresentation of SRF targets (curated transcription factor (TF) targets described in the TRANSFAC database) in 1,072 upregulated DEGs (*P* < 0.01) and 722 rd-DEGs (*P* < 0.01) (Fig. 5h), indicating that inhibition of PARP1 promoted SRF target transcripts.

Here, infection with Ad-PARP1 suppressed myocardin or myocardin–SRF overexpression-induced luciferase activities driven by WT-*SM22α* (Fig. 5i), indicating that overexpression of PARP1 suppressed myocardin–SRF-dependent promoter activation. Consistently, infection with Ad-PARP1 also suppressed myocardin or myocardin–SRF overexpression-induced 6×CArG-drove luciferase activity. Therefore, gain of PARP1 suppressed myocardin–SRF transactivation.

### Deletion of c-Jun abolished PARP1-induced VSMC proliferation and migration

**Fig. 6:**
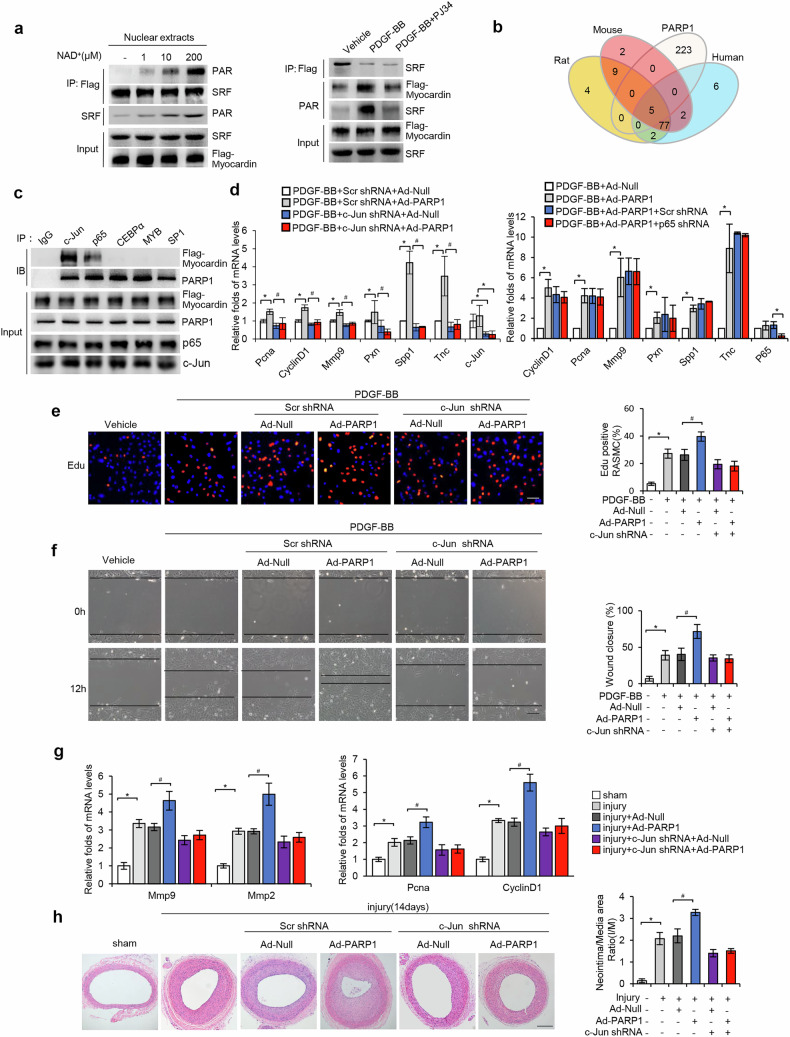
C-Jun mediates PARP1-induced VSMC proliferation and migration. **a**, Poly(ADP-ribosyl)ation and coprecipitates of myocardin and SRF in nuclear extracts of T/G-HAVSMCs infected with Flag-myocardin adenovirus, which were preincubated with active DNA and NAD^+^ (0, 1, 10 or 200 μM) (left); poly(ADP-ribosyl)ation and coprecipitates of Flag-myocardin and SRF in nuclear extracts from Flag-myocardin adenovirus infected rVSMCs treated with PDGF-BB or PDGF-BB + PJ34 for 48 h (right). **b**, A Venn diagram of PARP1-binding TFs whose binding sites exist in the promoters of *PCNA*, *CyclinD1*, *MMP2* and *MMP9* genes of human, mouse and rat. **c**, Coprecipitates of c-Jun, p65, CEBPα, MYB, SP1 with myocardin or PARP1 in nuclear extracts of T/G-HAVSMCs infected with Flag-myocardin adenovirus were determined using co-IP. **d**, rVSMCs were infected with Ad-c-Jun shRNA or Ad-p65 shRNA, together with Ad-Null or Ad-PARP1, and then treated with PDGF-BB for 24 h. The mRNA levels of *CyclinD1, Pcna*, *Mmp9*, *Pxn*, *Spp1* and *Tnc* genes were determined using qRT–PCR (*n* = 4). **e**, **f**, PDGF-BB-treated rVSMCs were infected with Ad-Scr shRNA or Ad-c-Jun shRNA, and then simultaneously infected with Ad-PARP1 or Ad-Null (*n* = 4): rVSMC proliferation was determined using EdU staining (scale bar, 20 μm) (**e**); rVSMC migration was determined using a wound healing assay (scale bar, 20 μm) (**f**). **g**, **h**, Balloon-injured rat arteries were in-site infected with Ad-Scr shRNA or Ad-c-Jun shRNA immediately after injury, together with Ad-Null or Ad-PARP1 (*n* = 5-8): mRNA levels of *Mmp9*, *Mmp2*, *Pcna* and *CyclinD1* in injured arteries at 10 days (**g**); H&E staining for neointima and the neointima/media ratio of injured arteries (scale bar, 50 μm) (**h**). Throughout, data are presented as mean ± s.d. *P < 0.05 versus control or PDGF-BB + Scr shRNA + Ad-Null or PDGF-BB + Ad-Null, ^#^*P* < 0.05 versus *P*DGF-BB + Scr shRNA + Ad-PARP1 or PDGF-BB + Ad-Null.

Many TFs have been reported to stimulate VSMC proliferation and migration through disrupting myocardin–SRF interaction^23–25^. Here, although incubation of nuclear extracts from T/G HA-VSMCs with NAD^+^/active DNA increased poly(ADP-ribosyl)ation of both SRF and myocardin, the amount of myocardin–SRF coprecipitates did not change (Fig. 6a). Moreover, treatment with PJ34 did not affect myocardin–SRF coprecipitates in the nuclear extracts from PDGF-BB-treated cells (Fig. 6a). Together, activation of PARP1 did not alter myocardin–SRF interaction, indicating alternative factors involved in the regulation of VSMC proliferation and migration.

TFBIND software was used to look for potential common TF-binding domain sites (TFBDs) in the promoters of *CyclinD1*, *PCNA*, *MMP9* and *MMP2* genes between rodents and human. In total, 84 common TFBDs were identified (Fig. 6b). Among corresponding TFs of these TFBDs, c-Jun, p65, C/EBPα, MYB and Sp1 have been reported to interact with PARP1^26–30^. As myocardin plays an important role in PARP1-induced VSMC proliferation and migration, we next investigated the TF(s) that interact with both PARP1 and myocardin. Co-IP showed that, among five PARP1-binding TFs, only P65 and c-Jun could coprecipitate with myocardin in the nuclear extracts from T/G HA-VSMCs (Fig. 6c), indicating that P65 and c-Jun could interact with both myocardin and PARP1, as previously reported^26,29,31,32^.

Infection with adenovirus encoding shRNA against c-Jun (shc-Jun), but not against p65 (shp65), abolished PARP1 overexpression-induced upregulation of *CyclinD1*, *Pcna, Mmp9*, *Pxn*, *Spp1* and *Tnc* genes (Fig. 6d), suggesting that c-Jun mediated the effects of PARP1 on VSMC proliferation and migration. In line with this, c-Jun knockdown in VSMCs abrogated PARP1 overexpression-induced EdU incorporation and wound closure (Fig. 6e, f). Moreover, infection with shc-Jun versus scramble shRNA abolished PARP1 overexpression-induced expression of *CyclinD1*, *Pcna*, *Mmp9* and *Mmp2* genes, as well as the increase in the neointima/media ratio of balloon-injured arteries (Fig. 6g, h). Together, c-Jun contributed critically to PARP1-induced VSMC proliferation and migration in vitro and in vivo.

### Activation of PARP1 abolished myocardin-induced suppression of c-Jun transactivation in VSMCs

**Fig. 7:**
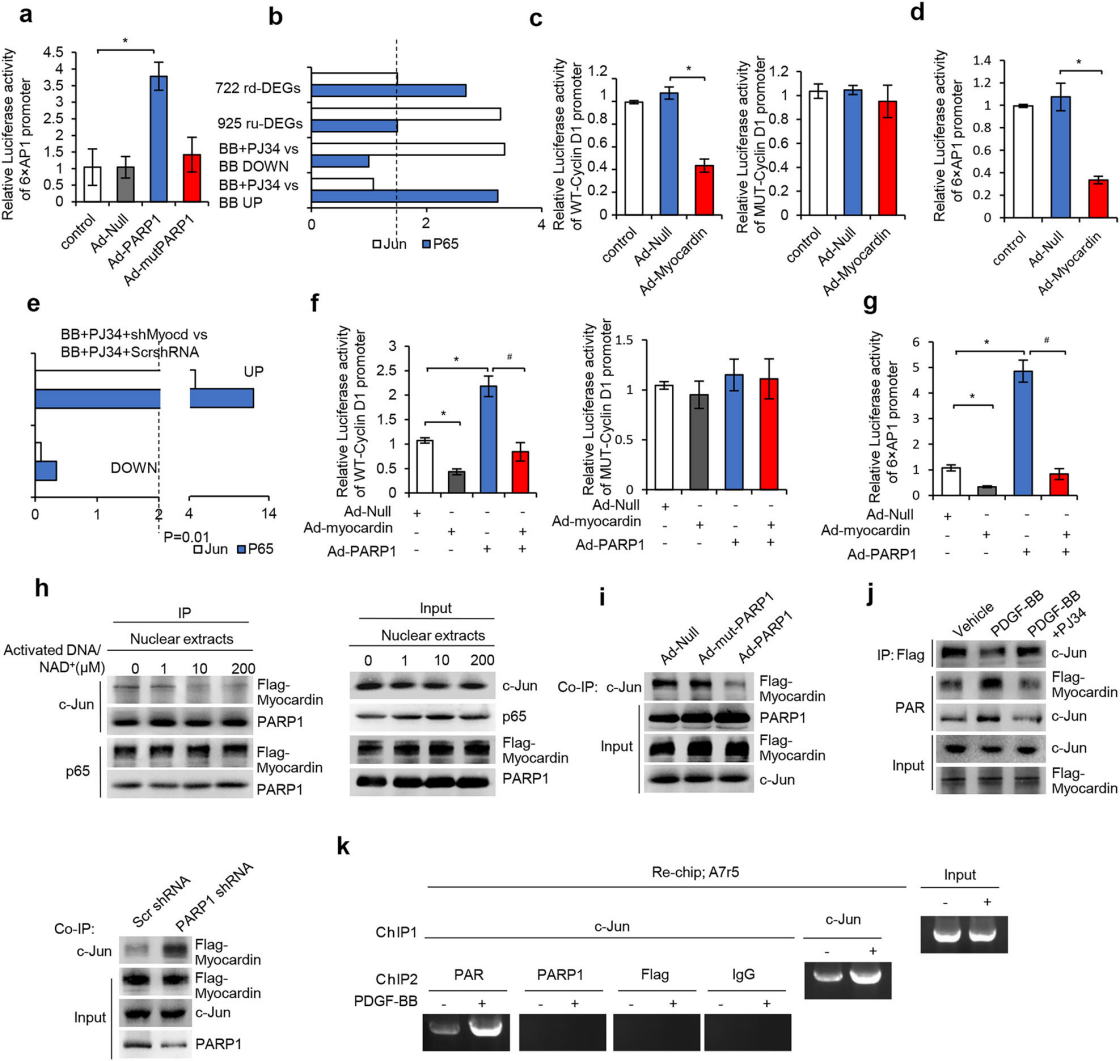
PARP1 abolishes myocardin-induced repression of c-Jun through poly(ADP-ribosyl)ation of both proteins. **a**, Luciferase activity driven by 6×AP1 reporter in A7r5 cells, which were infected with Ad-PARP1 or Ad-mut-PARP1 (enzymatic mutant) (*n* = 4). **b**, TF target gene sets were collected from the MSigDB C3 TFT motif gene sets, and multiple target gene sets of certain TF were combined to serve as an integrated targets source, including AP1: AP_1, AP_C, AP1_Q2, AP1_Q2_01, AP1_Q4, AP1_Q4_01, AP1_Q6, AP1_Q6_01, P65: NFKAPPAB65_01. Fisher’s exact test had been used to detect TF target overrepresentation in DEGs of vehicle, PDGF-BB and PDGF-BB + PJ34. **c**, **d**, Luciferase activity driven by WT or AP1-binding site mutant *CyclinD1* promoter (WT-*CyclinD1* and MUT-*CyclinD1*, respectively) (**c**), or 6×AP1 (**d**) in A7r5 cells infected with Ad-myocardin or Ad-Null (*n* = 4). **e**, TF target gene sets were collected from the MSigDB C3 TFT motif gene sets, and multiple target gene sets of certain TF were combined to serve as an integrated targets source, including AP1: AP_1, AP_C, AP1_Q2, AP1_Q2_01, AP1_Q4, AP1_Q4_01, AP1_Q6, AP1_Q6_01, P65: NFKAPPAB65_01. Fisher’s exact test had been used to detect TF target overrepresentation in DEGs of PDGF-BB + PJ34 + Scr and PDGF-BB + PJ34 + myocardin shRNA. **f**, **g**, A7r5 cells were infected with Ad-myocardin or Ad-PARP1, and the luciferase activity driven by WT-*CyclinD1* (left), MUT-*CyclinD1* (right) (**f**) or 6×AP1 (**g**) in A7r5 cells were determined at 48 h after reporter transfection (*n* = 4). **h**, Nuclear extracts from T/G-HAVSMCs infected with Flag-myocardin adenovirus were incubated with active DNA and NAD^+^ (0, 1, 10 or 200 μM). Coprecipitates of myocardin with c-Jun or p65, and coprecipitates of PARP1 with c-Jun or p65, were determined using co-IP assay. **i**, Coprecipitates of c-Jun and myocardin in nuclear extracts from Ad-PARP1 or Ad-mut-PARP1-infected T/G-HAVSMCs along with Flag-myocardin adenoviral transduction (*n* = 4). **j**, Coprecipitates of c-Jun and myocardin in nuclear extracts from Flag-myocardin-infected A7r5 cells infected with Ad-Scr shRNA or Ad-PARP1 shRNA (left); coprecipitates and poly(ADP-ribosyl)ation of c-Jun and myocardin in nuclear extracts from PDGF-BB-treated rVSMCs in the presence or absence of PJ34. **k**, A7r5 cells infected with Flag-myocardin adenovirus were treated with vehicle or PDGF-BB for 48 h. Chromatin was first immunoprecipitated with anti-c-Jun antibody, and then re-immunoprecipitated with anti-PAR, anti-PARP1, anti-Flag (myocardin) or anti-IgG antibody, respectively. Throughout, data are presented as mean ± s.d. *P < 0.05 versus Ad-Null, ^#^*P* < 0.05 versus Ad-PARP1.

In this study, infection with Ad-PARP1 increased luciferase activity driven by six tandem repeats of the AP1-binding site (6×AP1), while infection with adenovirus encoding a mutant PARP1 lacking enzymatic activity (Ad-mut-PARP1) did not (Fig. 7a), indicating that enzymatic activity was indispensable for PARP1-induced c-Jun transactivation. In line with this, enrichment analysis showed that treatment with PJ34 resulted in an overrepresentation of c-Jun targets in 1591 downregulated DEGs (*P* < 0.01) and 925 ru-DEGs (*P* < 0.01) (Fig. 7b), indicating that inhibition of PARP1 activity suppressed c-Jun target transcripts.

Myocardin is capable of suppressing VSMC proliferation^33^. Here, infection with Ad-myocardin versus Ad-Null suppressed the luciferase activities driven by WT, but not by AP1-binding site-mutated (MUT), promoters of *CyclinD1* and *MMP9* genes (targets of c-Jun, respectively) (Fig. 7c and Supplementary Fig. 7a), indicating that overexpression of myocardin suppressed c-Jun-dependent promoter activation. Consistently, infection with Ad-myocardin versus Ad-Null suppressed luciferase activity driven by 6×AP1 (Fig. 7d). Therefore, myocardin suppressed c-Jun transactivation in VSMCs. Consistently, enrichment analysis showed that knockdown of myocardin led to overrepresentation of c-Jun targets in 2903 upregulated DEGs (*P* < 0.01) (Fig. 7e).

Now that PARP1 promoted c-Jun activation, we suspected that it might abolish myocardin-induced c-Jun suppression. This was confirmed by the fact that infection with Ad-PARP1 reversed myocardin overexpression-induced reduction in luciferase activities driven by WT-*CyclinD1*, WT-*MMP9* promoter and 6×AP1 (Figs. 7f, g and Supplementary Fig. 7b).

### PARP1 disrupted myocardin–c-Jun interaction through poly(ADP-ribosyl)ation of both proteins

Both c-Jun and myocardin could be poly(ADP-ribosyl)ated by PARP1. Incubation of nuclear extracts from T/G HA-VSMCs with NAD^+^/active DNA, which enhanced endogenous poly(ADP-ribosyl)ation of myocardin and c-Jun, suppressed myocardin–c-Jun coprecipitates but did not affect myocardin–p65 coprecipitates (Fig. 7h), indicating that poly(ADP-ribosyl)ation suppressed myocardin–c-Jun interaction. In support of this, infection with Ad-PARP1 decreased myocardin–c-Jun coprecipitates, but infection with Ad- mut-PARP1 did not (Fig. 7i). Moreover, PDGF-BB-induced decreases in myocardin–c-Jun coprecipitates were reversed when PARP1 was knocked down or inhibited by PJ34 (Fig. 7j). Together, PARP1 disrupted myocardin–c-Jun interaction through poly(ADP-ribosyl)ation of both proteins. Moreover, re-ChIP showed that poly(ADP-ribosyl)ated c-Jun could bind to the cyclinD1 promoter in A7r5-VSMCs. Myocardin, neither poly(ADP-ribosyl)ated nor un-poly(ADP-ribosyl)ated, could not be recruited to the cyclinD1 promoter (Fig. 7k). Together, myocardin and c-Jun could not interact with each other after poly(ADP-ribosyl)ation.

## Discussion

Excessive VSMC proliferation, migration and secretion of various extracellular matrix proteins and cytokines is only the hallmark feature but also the driving force of neointimal hyperplasia and atherosclerosis^1,34^. Here, we observe a robust activation of PARP1 in VSMCs of artery stenosis and atherosclerotic plaques in rodents and humans. Moreover, PARP1 is identified as a critical factor in defining VSMC phenotype. Mechanistically, PARP1 binds to and poly(ADP-ribosy)lates myocardin–SRF to suppress contractile protein expression, while poly(ADP-ribosy)lated myocardin relieves the inhibitory effects on c-Jun, thus promoting proliferation and migration of VSMCs.

In eukaryocytes, PARP1 functions at the center of cellular stress responses, where it senses and integrates various stress signals through poly(ADP-ribosyl)ation of diverse acceptor proteins, deciding the fate of cells on the basis of the type and strength of the stimulus^5^. Here, we show that PARP1 is robustly and sustainedly activated in VSMCs of artery stenosis and atherosclerotic plaques. Moreover, pharmacological or genetic manipulation of PARP1 alters the phenotype of VSMCs in vivo and in vitro, highlighting the critical role for PARP1 in regulating VSMC phenotype. These findings illustrate that PARP1 should be a novel potential therapeutic target for vascular diseases including artery stenosis and atherosclerosis.

In VSMCs, contractile gene expression is controlled by the myocardin–SRF complex^19^. In this complex, SRF directly binds to CArG motifs in the target promoters. However, SRF alone is insufficient to initiate transcription; it needs to associate with myocardin to fasten the promoter binding and facilitate transcription. Many TFs, including YAP, ELK-1, HERP1, KLF4, P65, c-Jun and Runx2, are capable of competitively interacting with myocardin or SRF to disrupt myocardin–SRF interaction and thereby suppress contractile gene expression^23–25,31,32,35,36^. However, we showed here that PARP1-induced suppression of contractile genes is not mediated through disrupting myocardin–SRF interaction, because the interaction does not change after poly(ADP-ribosyl)ation. Instead, we find that this suppression is mediated through poly(ADP-ribosyl)ation of myocardin and SRF per se. Poly(ADP-ribosyl)ation is an important posttranslational modification capable of altering the DNA-binding capacity of acceptor proteins through two distinct mechanisms: on the one hand, addition of negative charged poly(ADP-ribose) polymer causes negative charge repulsion between acceptor protein and DNA strands, leading to decreased DNA binding; on the other hand, poly(ADP-ribose) polymers might form a DNA-binding scaffold on the acceptor protein to facilitate its DNA binding^37^. Here, poly(ADP-ribosyl)ation dramatically decreases the DNA-binding capacity of the myocardin–SRF complex, indicating that addition of poly(ADP-ribose) polymers causes a negative charge repulsion between the complex rather than forming a DNA-binding scaffold. Moreover, because myocardin is indispensable for PARP1-mediated poly(ADP-ribosyl)ation of SRF, this finding highlights the pivotal role for myocardin in PARP1-induced contractile gene suppression.

Myocardin is capable of suppressing VSMC proliferation and migration under basal conditions. Long et al. report that myocardin suppresses VSMC proliferation through induction of miR-24 and miR-29a^38^. However, here, inhibition of PARP1 did not affect miR-24 and miR-29a expression (Supplementary Fig. 8). Tang et al. also report that myocardin suppresses cell proliferation through interaction with p65^31^. However, the effects of p65 on VSMC proliferation are controversial^39,40^. Moreover, we show that p65 is not involved in the PARP1-induced phenotype switch. In contrast to p65, we find that c-Jun mediates the effects of PARP1 on VSMC proliferation and migration. Further study reveals that myocardin can dramatically repress c-Jun transactivity through interaction with it. Given that c-Jun has been well demonstrated to promote VSMC proliferation and neointima formation after arterial injury^41,42^, this finding, from another point of view, uncovers an important mechanism by which myocardin suppresses VSMC proliferation and migration under basal conditions, when it is highly expressed.

Here, we demonstrate that poly(ADP-ribosyl)ation of myocardin and c-Jun disrupts their interaction and, thus, abrogates myocardin-induced repression of c-Jun. Commonly, poly(ADP-ribosyl)ation affects protein–protein interaction via different manners: (1) inhibiting the interaction through masking protein–protein interaction sites or introducing charge repulsion with strongly negatively charged polymer; (2) enhancing the interaction through forming a PAR-based scaffold on acceptor proteins, which recruits PAR-binding proteins to their sites of action^37^. Here, myocardin and c-Jun interact with each other under basal conditions, but not after poly(ADP-ribosyl)ation, indicating that addition of PAR-polymer suppresses the interaction between them. Taken together, activation of PARP1 abrogates myocardin-induced repression of c-Jun, promoting c-Jun transactivation and gene expression, stimulating VSMC proliferation and phenotypic transformation.

In this study, poly(ADP-ribosyl)ation does not affect myocardin–SRF interaction, but disrupts myocardin–c-Jun interaction. Moreover, the effect of poly(ADP-ribosyl)ation on the DNA-binding capacity of myocardin–SRF also differs from its effect on the binding of c-Jun to DNA. These discrepancies, although reflecting the complexity of poly(ADP-ribosyl)ation in the regulation of different acceptor proteins, synthetically promote VSMC phenotypic transformation, thus highlighting the central role of PARP1 activation in the orchestration of VSMC phenotype switch. All these findings indicate that inhibition of PARP1 activation could be a novel potential therapeutic strategy against vascular diseases, including artery stenosis and coronary heart diseases.

## Supplementary information

Supplementary Information

## References

[1] Owens, G. K., Kumar, M. S. & Wamhoff, B. R. Molecular regulation of vascular smooth muscle cell differentiation in development and disease. *Physiol. Rev.* **84**, 767–801 (2004). 15269336 10.1152/physrev.00041.2003

[2] Gomez, D. & Owens, G. K. Reconciling smooth muscle cell oligoclonality and proliferative capacity in experimental atherosclerosis. *Circ. Res.* **119**, 1262–1264 (2016). 27932466 10.1161/CIRCRESAHA.116.310104 PMC5157924

[3] Chappell, J. et al. Extensive proliferation of a subset of differentiated, yet plastic, medial vascular smooth muscle cells contributes to neointimal formation in mouse injury and atherosclerosis models. *Circ. Res.* **119**, 1313–1323 (2016). 27682618 10.1161/CIRCRESAHA.116.309799 PMC5149073

[4] Kim, M. Y., Zhang, T. & Kraus, W. L. Poly(ADP-ribosyl)ation by PARP-1: ‘PAR-laying’ NAD+ into a nuclear signal. *Genes Dev.* **19**, 1951–1967 (2005). 16140981 10.1101/gad.1331805

[5] Luo, X. & Kraus, W. L. On PAR with PARP: cellular stress signaling through poly(ADP-ribose) and PARP-1. *Genes Dev.* **26**, 417–432 (2012). 22391446 10.1101/gad.183509.111 PMC3305980

[6] Zhang, H., Xiong, Z. M. & Cao, K. Mechanisms controlling the smooth muscle cell death in progeria via down-regulation of poly(ADP-ribose) polymerase 1. *Proc. Natl Acad. Sci. USA* **111**, E2261–E2270 (2014). 24843141 10.1073/pnas.1320843111 PMC4050581

[7] Rom, S. et al. PARP inhibition in leukocytes diminishes inflammation via effects on integrins/cytoskeleton and protects the blood–brain barrier. *J. Neuroinflammation* **13**, 254 (2016). 27677851 10.1186/s12974-016-0729-x PMC5039899

[8] Ba, X. & Garg, N. J. Signaling mechanism of poly(ADP-ribose) polymerase-1 (PARP-1) in inflammatory diseases. *Am. J. Pathol.* **178**, 946–955 (2011). 21356345 10.1016/j.ajpath.2010.12.004 PMC3069822

[9] Mathews, M. T. & Berk, B. C. PARP-1 inhibition prevents oxidative and nitrosative stress-induced endothelial cell death via transactivation of the VEGF receptor 2. *Arterioscler Thromb. Vasc. Biol.* **28**, 711–717 (2008). 18239155 10.1161/ATVBAHA.107.156406

[10] Pacher, P. & Szabo, C. Role of poly(ADP-ribose) polymerase 1 (PARP-1) in cardiovascular diseases: the therapeutic potential of PARP inhibitors. *Cardiovasc. Drug Rev.* **25**, 235–260 (2007). 17919258 10.1111/j.1527-3466.2007.00018.x PMC2225457

[11] Wang, C. et al. Poly(ADP-ribose) polymerase 1 accelerates vascular calcification by upregulating Runx2. *Nat. Commun.* **10**, 1203 (2019). 30867423 10.1038/s41467-019-09174-1 PMC6416341

[12] Tulis, D. A. Rat carotid artery balloon injury model. *Methods Mol. Med.* **139**, 1–30 (2007). 18287662 10.1007/978-1-59745-571-8_1 PMC2819386

[13] Wang, J. N., Shi, N., Xie, W. B., Guo, X. & Chen, S. Y. Response gene to complement 32 promotes vascular lesion formation through stimulation of smooth muscle cell proliferation and migration. *Arterioscler. Thromb. Vasc. Biol.* **31**, e19–e26 (2011). 21636805 10.1161/ATVBAHA.111.230706 PMC3146015

[14] Ballantyne, M. D. et al. Smooth muscle enriched long noncoding RNA (SMILR) regulates cell proliferation. *Circulation* **133**, 2050–2065 (2016). 27052414 10.1161/CIRCULATIONAHA.115.021019 PMC4872641

[15] Wang, C. et al. Poly(ADP-ribose) polymerase 1 promotes oxidative-stress-induced liver cell death via suppressing farnesoid X receptor alpha. *Mol. Cell Biol.* **33**, 4492–4503 (2013). 24043304 10.1128/MCB.00160-13 PMC3838191

[16] Xu, W. et al. A20 prevents obesity-induced development of cardiac dysfunction. *J. Mol. Med.* **96**, 159–172 (2018). 29143862 10.1007/s00109-017-1608-3

[17] Mack, C. P., Thompson, M. M., Lawrenz-Smith, S. & Owens, G. K. Smooth muscle alpha-actin CArG elements coordinate formation of a smooth muscle cell-selective, serum response factor-containing activation complex. *Circ. Res.* **86**, 221–232 (2000). 10666419 10.1161/01.res.86.2.221

[18] Gupte, R., Liu, Z. & Kraus, W. L. PARPs and ADP-ribosylation: recent advances linking molecular functions to biological outcomes. *Genes Dev.* **31**, 101–126 (2017). 28202539 10.1101/gad.291518.116 PMC5322727

[19] Wang, Z., Wang, D. Z., Pipes, G. C. & Olson, E. N. Myocardin is a master regulator of smooth muscle gene expression. *Proc. Natl Acad. Sci. USA* **100**, 7129–7134 (2003). 12756293 10.1073/pnas.1232341100 PMC165841

[20] Wang, Z. et al. Myocardin and ternary complex factors compete for SRF to control smooth muscle gene expression. *Nature* **428**, 185–189 (2004). 15014501 10.1038/nature02382

[21] Pipes, G. C., Creemers, E. E. & Olson, E. N. The myocardin family of transcriptional coactivators: versatile regulators of cell growth, migration, and myogenesis. *Genes Dev.* **20**, 1545–1556 (2006). 16778073 10.1101/gad.1428006

[22] McDonald, O. G., Wamhoff, B. R., Hoofnagle, M. H. & Owens, G. K. Control of SRF binding to CArG box chromatin regulates smooth muscle gene expression in vivo. *J. Clin. Invest.* **116**, 36–48 (2006). 16395403 10.1172/JCI26505 PMC1323266

[23] Tanaka, T. et al. Runx2 represses myocardin-mediated differentiation and facilitates osteogenic conversion of vascular smooth muscle cells. *Mol. Cell Biol.* **28**, 1147–1160 (2008). 18039851 10.1128/MCB.01771-07 PMC2223399

[24] Doi, H. et al. HERP1 inhibits myocardin-induced vascular smooth muscle cell differentiation by interfering with SRF binding to CArG box. *Arterioscler. Thromb. Vasc. Biol.* **25**, 2328–2334 (2005). 16151017 10.1161/01.ATV.0000185829.47163.32

[25] Xie, C. et al. Yap1 protein regulates vascular smooth muscle cell phenotypic switch by interaction with myocardin. *J. Biol. Chem.* **287**, 14598–14605 (2012). 22411986 10.1074/jbc.M111.329268 PMC3340286

[26] Huang, D., Wang, Y., Yang, C., Liao, Y. & Huang, K. Angiotensin II promotes poly(ADP-ribosyl)ation of c-Jun/c-Fos in cardiac fibroblasts. *J. Mol. Cell. Cardiol.* **46**, 25–32 (2009). 19027749 10.1016/j.yjmcc.2008.10.019

[27] Yin, H. & Glass, J. In prostate cancer cells the interaction of C/EBPα with Ku70, Ku80, and poly(ADP-ribose) polymerase-1 increases sensitivity to DNA damage. *J. Biol. Chem.* **281**, 11496–11505 (2006). 16490787 10.1074/jbc.M511138200

[28] Cervellera, M. N. & Sala, A. Poly(ADP-ribose) polymerase is a B-MYB coactivator. *J. Biol. Chem.* **275**, 10692–10696 (2000). 10744766 10.1074/jbc.275.14.10692

[29] Kameoka, M. et al. Evidence for regulation of NF-kappaB by poly(ADP-ribose) polymerase. *Biochem. J.* **346**, 641–649 (2000). 10698690 PMC1220896

[30] Zaniolo, K., Desnoyers, S., Leclerc, S. & Guerin, S. L. Regulation of poly(ADP-ribose) polymerase-1 (PARP-1) gene expression through the post-translational modification of Sp1: a nuclear target protein of PARP-1. *BMC Mol. Biol.* **8**, 96 (2007). 17961220 10.1186/1471-2199-8-96 PMC2175517

[31] Tang, R. H. et al. Myocardin inhibits cellular proliferation by inhibiting NF-κB(p65)-dependent cell cycle progression. *Proc. Natl Acad. Sci. USA* **105**, 3362–3367 (2008). 18296632 10.1073/pnas.0705842105 PMC2265140

[32] Gordon, J. W. et al. Protein kinase A-regulated assembly of a MEF2·HDAC4 repressor complex controls c-Jun expression in vascular smooth muscle cells. *J. Biol. Chem.* **284**, 19027–19042 (2009). 19389706 10.1074/jbc.M109.000539 PMC2707197

[33] Long, X. & Miano, J. M. Myocardin: new therapeutic agent in vascular disease?. *Arterioscler. Thromb. Vasc. Biol.* **33**, 2284–2285 (2013). 24025544 10.1161/ATVBAHA.113.302068 PMC3894635

[34] Wang, J. et al. Hierarchical capillary coating to biofunctionlize drug-eluting stent for improving endothelium regeneration. *Research* **2020**, 1458090 (2020). 32885169 10.34133/2020/1458090 PMC7455884

[35] Liu, Y. et al. Kruppel-like factor 4 abrogates myocardin-induced activation of smooth muscle gene expression. *J. Biol. Chem.* **280**, 9719–9727 (2005). 15623517 10.1074/jbc.M412862200

[36] Taurin, S. et al. Phosphorylation of myocardin by extracellular signal-regulated kinase. *J. Biol. Chem.* **284**, 33789–33794 (2009). 19776005 10.1074/jbc.M109.048983 PMC2797148

[37] Ryu, K. W., Kim, D. S. & Kraus, W. L. New facets in the regulation of gene expression by ADP-ribosylation and poly(ADP-ribose) polymerases. *Chem. Rev.* **115**, 2453–2481 (2015). 25575290 10.1021/cr5004248 PMC4378458

[38] Talasila, A. et al. Myocardin regulates vascular response to injury through miR-24/-29a and platelet-derived growth factor receptor-beta. *Arterioscler. Thromb. Vasc. Biol.* **33**, 2355–2365 (2013). 23825366 10.1161/ATVBAHA.112.301000

[39] Autieri, M. V., Yue, T. L., Ferstein, G. Z. & Ohlstein, E. Antisense oligonucleotides to the p65 subunit of NF-kB inhibit human vascular smooth muscle cell adherence and proliferation and prevent neointima formation in rat carotid arteries. *Biochem. Biophys. Res. Commun.* **213**, 827–836 (1995). 7654244 10.1006/bbrc.1995.2204

[40] Mehrhof, F. B., Schmidt-Ullrich, R., Dietz, R. & Scheidereit, C. Regulation of vascular smooth muscle cell proliferation: role of NF-κB revisited. *Circ. Res.* **96**, 958–964 (2005). 15831813 10.1161/01.RES.0000166924.31219.49

[41] Khachigian, L. M., Fahmy, R. G., Zhang, G., Bobryshev, Y. V. & Kaniaros, A. c-Jun regulates vascular smooth muscle cell growth and neointima formation after arterial injury. Inhibition by a novel DNA enzyme targeting c-Jun. *J. Biol. Chem.* **277**, 22985–22991 (2002). 11891228 10.1074/jbc.M200977200

[42] Shaulian, E. & Karin, M. AP-1 in cell proliferation and survival. *Oncogene* **20**, 2390–2400 (2001). 11402335 10.1038/sj.onc.1204383

