# Supplementary material for: Poly(ADP-ribose) polymerase 1 orchestrates vascular smooth muscle cell homeostasis in arterial disease
Source: Exp Mol Med. 2025 Aug 1;57(8):1686–99. doi: 10.1038/s12276-025-01501-5 (PMC12411612; doi:10.1038/s12276-025-01501-5)
Supplement: Supplementary file 1 — Supplementary Information [file 12276_2025_1501_MOESM1_ESM.pdf]

Supplementary Materials

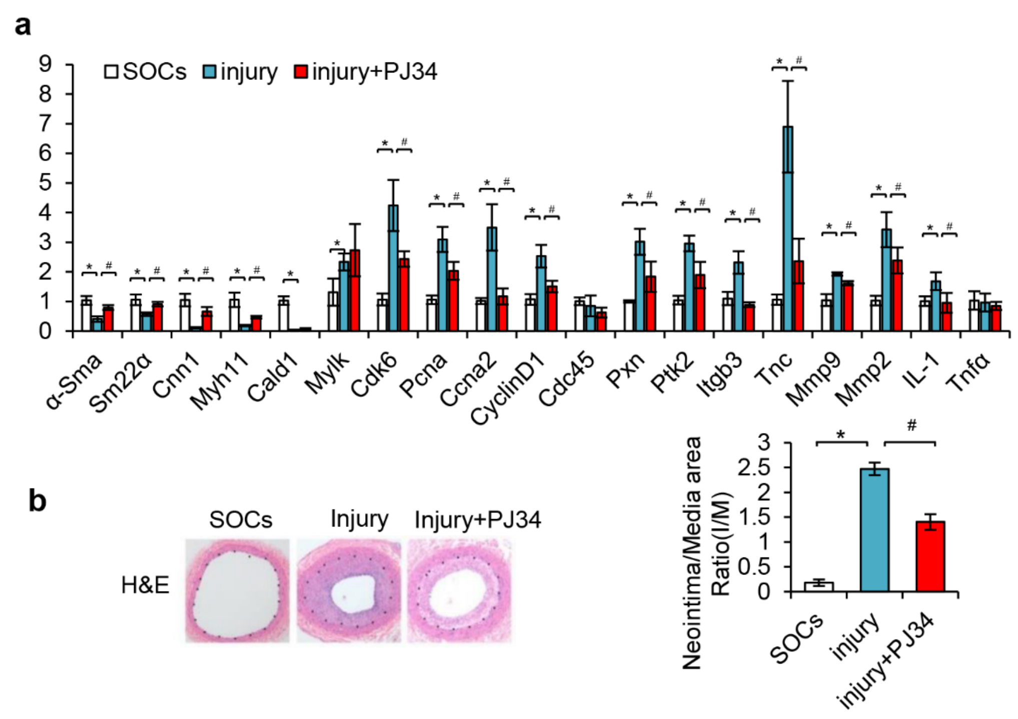

Supplementary Fig. 1: PARP inhibitor, PJ34 contributes importantly to VSMCs phenotype switch in vivo. **a** and **b**, After balloon injury, rats were randomly received intraperitoneal injection of PJ34 (10mg/kg/d) or Vehicle once a day for 14 days (n=5-8). The mRNA levels of genes involved in contraction, proliferation and migration were determined using qRT-PCR (upper), and H&E staining for neointima and the neointima/media ratio (bottom). The black spots indicated inner elastic discs.

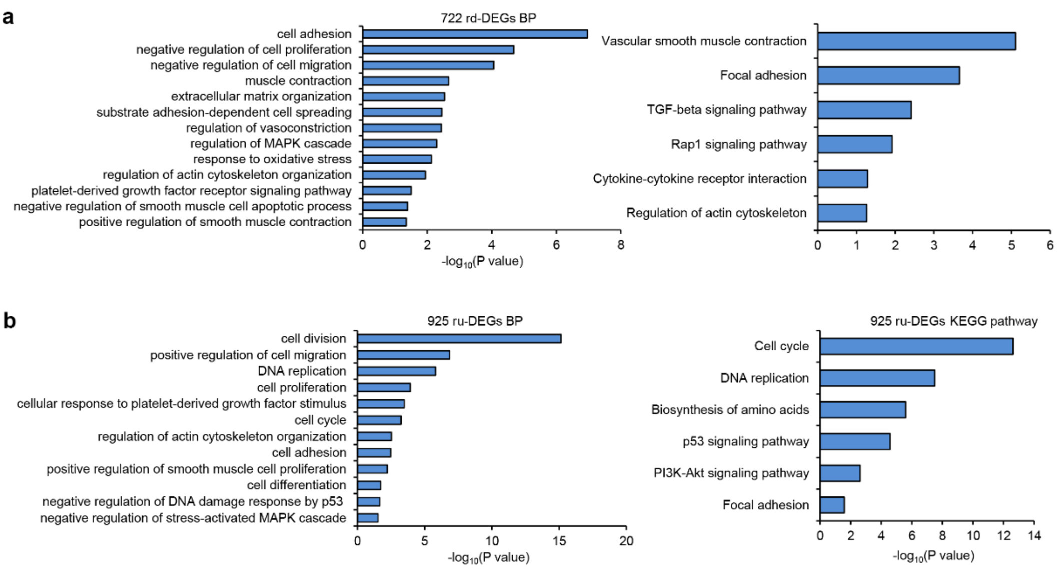

Supplementary Fig. 2: Functional category enrichment analysis. **a** and **b**, Primary rVSMCs were treated with Vehicle, PDGF-BB or PDGF-BB+PJ34 for 48 hours. The gene expression profiles were analyzed by RNA-Seq. Gene ontology (GO) and KEGG pathways analysis for the 722 rd-DEGs (**a**) and 925 ru-DEGs (**b**).

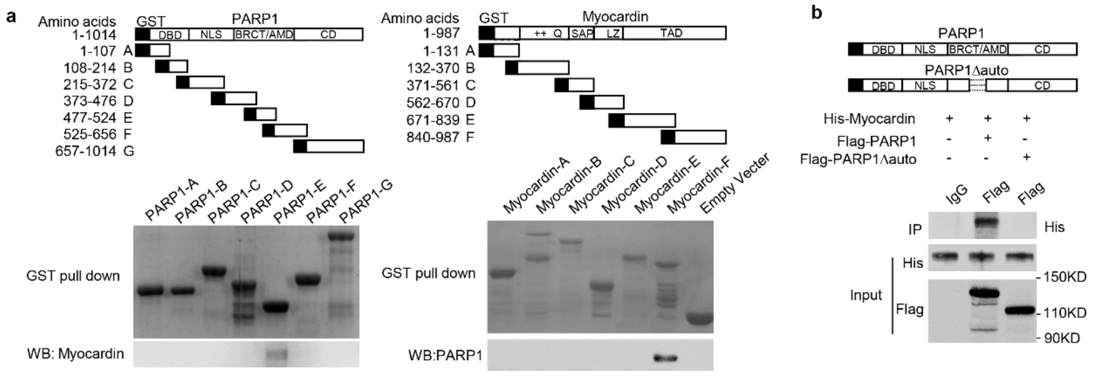

Supplementary Fig. 3: Mapping the interaction domain of PARP1 with myocardin. **a**, GST-PARP1 (upper left) and GST-myocardin (upper right) fragments were constructed, purified and detected by Coomassie staining (middle). GST pull-down assays were performed, followed by immunoblot with anti-myocardin (bottom left) or anti-PARP1(bottom right) antibody. **b**, Schematic representation of the domain deletion constructs of PARP1 (upper). 293T cells were transfected with the indicated combinations of constructs. Coprecipitates of Flag-tagged PARP1 and His-tagged myocardin in the nuclear extracts were determined using immunoblot (n=3).

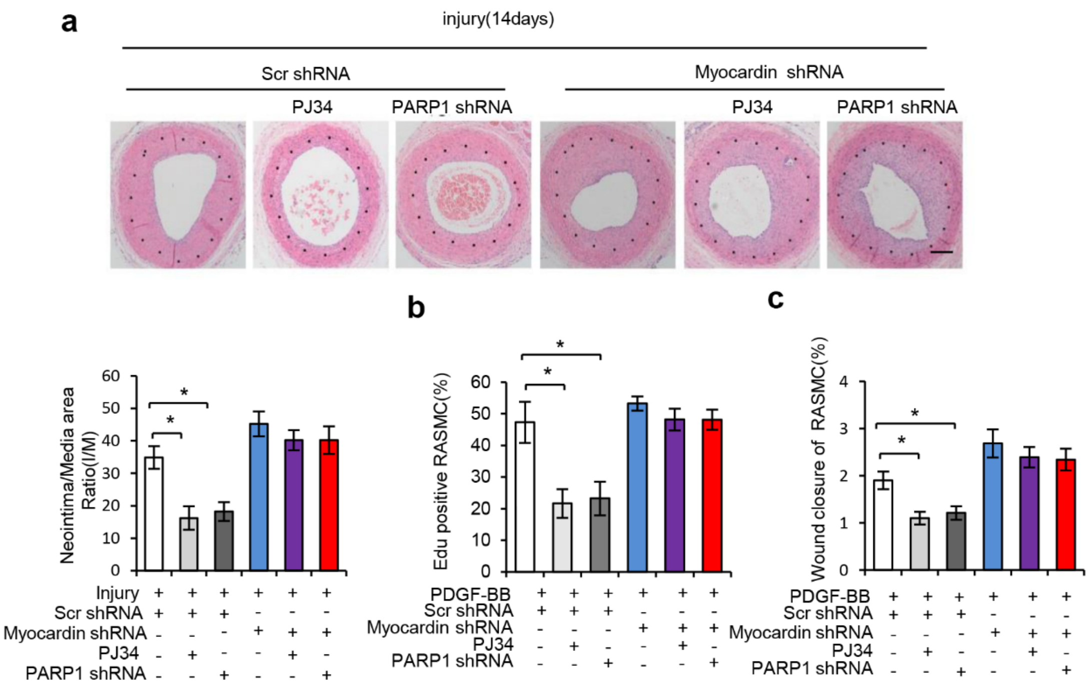

Supplementary Fig. 4: Myocardin mediates the effects of PARP1 inhibition on VSMC phenotype switch. **a**, Balloon-injured rat arteries were in-site infected with Ad-Scr shRNA or adenoviruses encoding myocardin shRNA (Ad-myocardin shRNA) immediately after injury, and then were treated with PJ34 or simultaneously infected with Ad-PARP1 shRNA for 10 days. (n=5-8). H&E staining was performed for neointima and the neointima/media ratio (bottom) was quantified. Scale bar=50 μm. **b** and **c**, PDGF-BB-treated rVSMCs were infected with Ad-Scr shRNA or Ad-myocardin shRNA, and then were treated with PJ34 or simultaneously infected with Ad-PARP1 (n=4). VSMC proliferation was determined using EdU staining (**b**) VSMC migration was determined using wound healing assay (**c**).

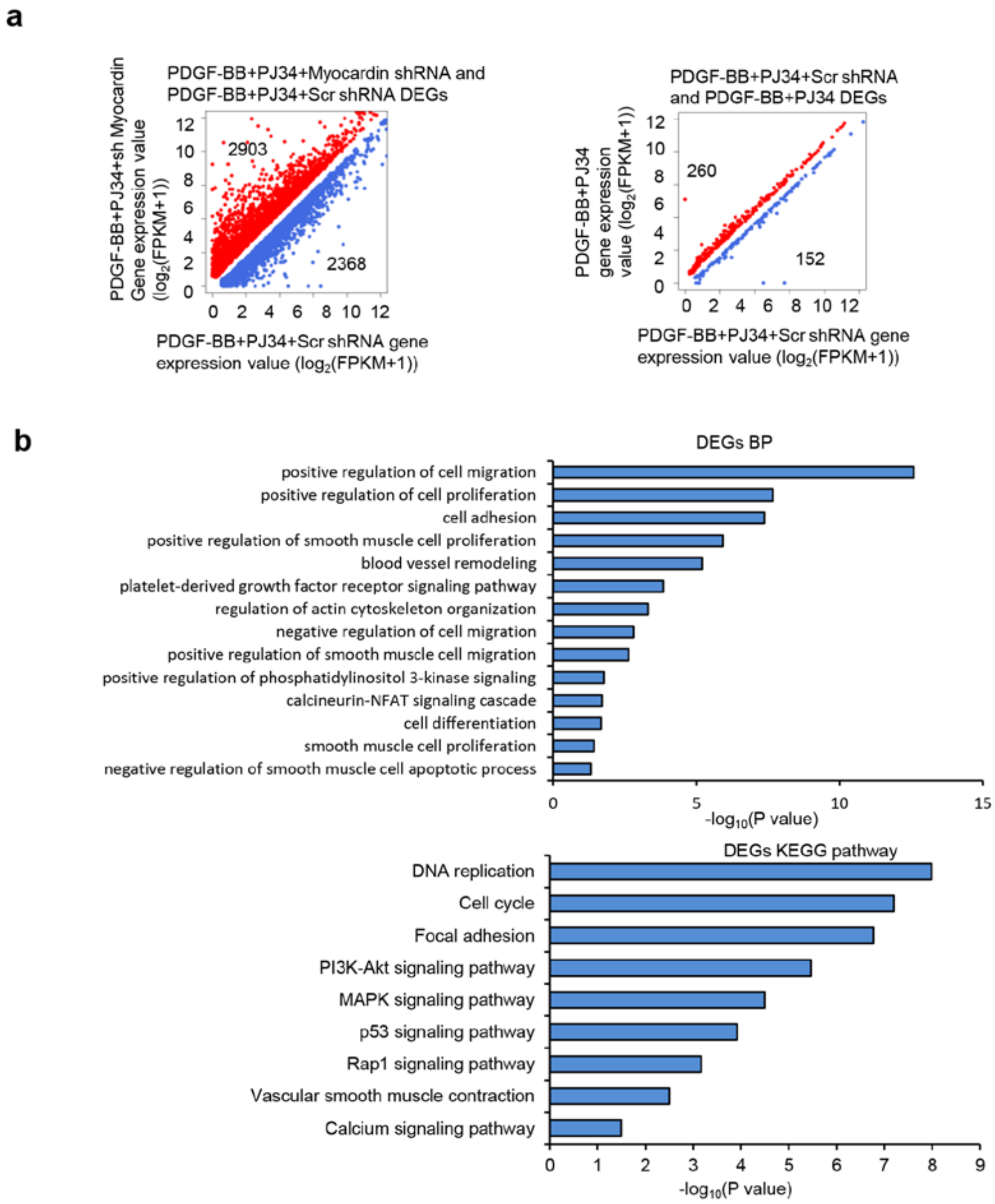

Supplementary Fig. 5. The effects of myocardin deletion on DEGs of VSMCs treated with both PDGF-BB and PJ34 and functional category enrichment analysis. **a** and **b**, Rat VSMCs were infected with Ad-myocardin shRNA or Ad-Scr shRNA, and then treated with PDGF-BB+PJ34 for 48 hours. The gene expression profiles were analyzed by RNA-Seq. **a**, Scatter plots of DEGs as indicated. Red dots represent related higher DEGs, and blue dots represent lower DEGs. **b**, GO terms and KEGG pathways related to DEGs between BB+PJ34+Scr shRNA and BB+ PJ34 +myocardin shRNA group.

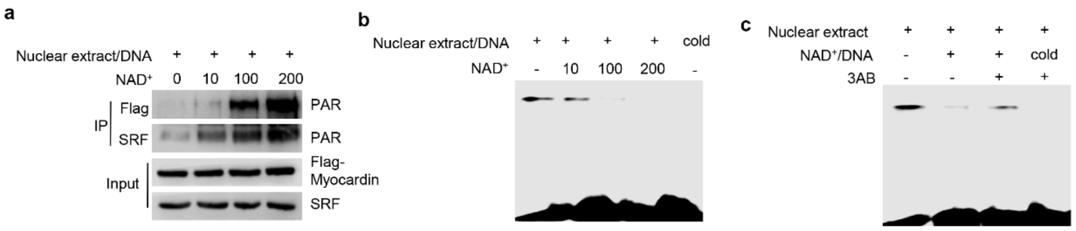

Supplementary Fig. 6: Poly(ADP-ribosyl)ation suppresses binding of myocardin/SRF to αSMA-CArG probe. **a** and **b**, Nuclear extracts from T/G-HAVSMCs infected with Flag-Myocardin adenovirus were incubated with active DNA and NAD+ (0, 10, 100, or 200 uM). **a**, Poly(ADP-ribosyl)ation of myocardin and SRF in nuclear extracts was determined by immunoblot. **b**, Binding of myocardin/SRF to αSMA-CArG probe was determined using EMSA assay. **c**, Nuclear extracts from T/G-HAVSMCs were incubated with active DNA/NAD+ alone or together with 3AB. Binding of myocardin/SRF to αSMA-CArG probe was determined using EMSA assay.

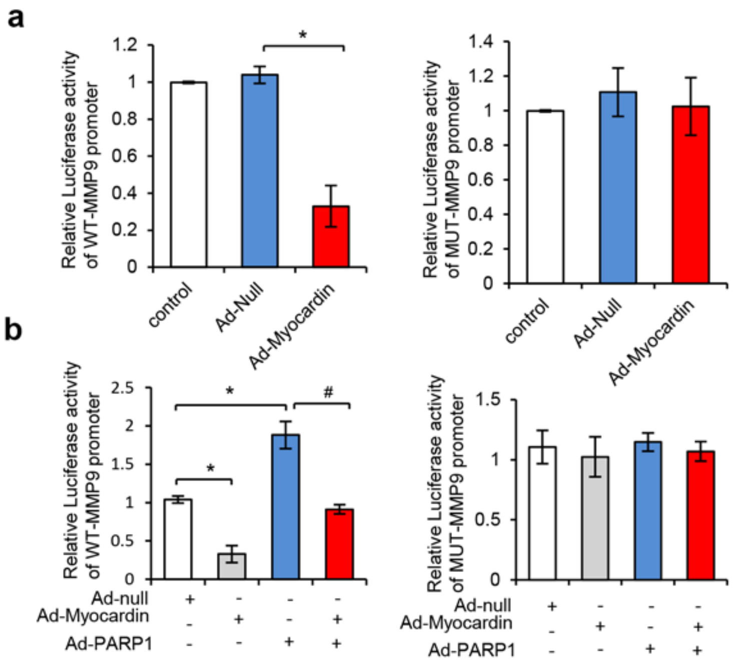

Supplementary Fig. 7: The effects of myocardin overexpression on the luciferase activities driven by MMP9 promoter. **a**, Luciferase activity driven by wild type or AP1 binding site mutant MMP9 promoter (WT-MMP9 and MUT-MMP9, respectively) in Ad-myocardin- or Ad-Null-infected A7r5 cells (n=4). **b**, Luciferase activity driven by WT-MMP9 and MUT-MMP9 promoter in A7r5 cells infected with Ad-myocardin, Ad-PARP1, or Ad-Null (n=4).

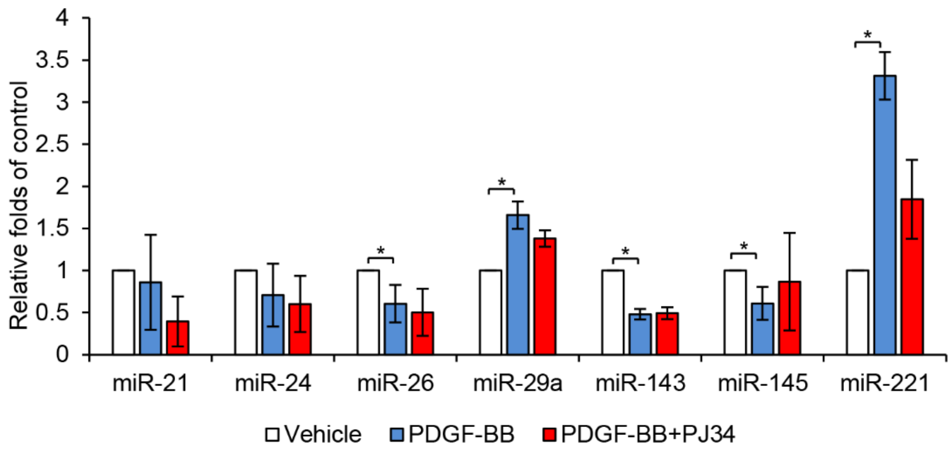

Supplementary Fig. 8: The effects of PJ34 on the expression of microRNAs in PDGF-BB-treated VSMCs. Primary rVSMCs were treated with Vehicle, PDGF-BB, or PDGF-BB+PJ34 for 24 hours. The expressions levels of miR-21, 24, 26, 29a, 143,145 and 221 were determined by qRT-PCR. (n=4). Throughout, data are presented as mean±s.d. *$P < 0.05$.

Supplementary Table 1

Primers Used for qRT-PCR

Gene Name	Forward Primer	Reverse Primer
PARP1 **(Rat)**	CTGTGAACTCCTCTGCACCA	AGCTGAGGCAGACACATCCT
P65 **(Rat)**	ATGGACGATCTGTTTCCC	GTCTTAGTGGTATCTGTGCT
α-Sma **(Rat)**	CACGAAACCACCTATAACAGCA	GAATATTTGCGTTCTGGAGGAG
Sm22 **(Rat)**	CATGTTCCAGACTGTTGACCTC	TGAACTCCCTCTTATGCTCCTG
Cnn1 **(Rat)**	GGGGTCAAATATGCAGAGAAAC	CTGCTGACTGGCAAACTTATTG
Myh11 **(Rat)**	GAAAGGAAACACCAAGGTCAAG	CTCACTGCGAAGTTTCTTGTG
Cald1 **(Rat)**	TGGAGAATTCATGACCCACA	AGCTCCTCCAGCCTCTTACC
Mylk **(Rat)**	GACGTGTTCACCCTGGTTCT	TTTGTGCAGCATCAGTGACA
Gapdh **(Rat)**	CCTCGTCTCATAGACAAGATGGT	CGTGGGTAGAGTCATACTGGAA
R18s **(Rat)**	GCAATTATTCCCCATGAACG	GGCCTCACTAAACCATCCAA
Cdk6 **(Rat)**	CAACGTGGTCAGGTTGTTTG	CACCGGGTTCTGGAACTTTA

Pcna **(Rat)**	GAACAGGAGTACAGCTGCGT	CTCCCCACTCGCAGAAAACT
Ccna2 **(Rat)**	ATGTCAACCCCGAAAAAGTG	GGGACGTGCTCATCGTTTAT
CyclinD1 **(Rat)**	ATTGCGGCTGGCTTTACATTAG	GCCTTCATTTGATCTGGGACAT
Cdc45 **(Rat)**	TCTAGAGCTCGCCAAGAAGC	TCGAGCACACAAAGGACTTG
Mmp2 **(Rat)**	CCATCGAGACCATGCGGAAG	CCTGTATGTGATCTGGTTCTTG
Mmp9 **(Rat)**	TTCGACGCTGACAAGAAGTG	AGGGGAGTCCTCGTGGTAGT
Pxn **(Rat)**	AGTGTGGAGAGCCTCTTGGA	CTGGGCCATGAACTTGAAAT
Ptk2 **(Rat)**	CTAAAAGCTCAGCTCAGCACAA	GCTGGGATAAAATCCTTCACTG
Itgb3 **(Rat)**	GACCACAGTGGGAGTCCTGT	CAACGATGAGCTGAAGGACA
Tnc **(Rat)**	CAGAAGCCTTGGCCATGTG	GCACTCTCTCCCCTGTGTAGGA
Tnf-α **(Rat)**	CTCAAAACTCGAGTGACAAGC	CCGTGATGTCTAAGTACTTGG
IL-1β **(Rat)**	GTGATGTTCCCATTAGACAGC	CTTTCATCACACAGGACAGG
Spp1 **(Rat)**	CCAAGCGTGGAAACACACAGCC	GGCTTTGGAACTCGCCTGACTG
α-Sma	GTCCCAGACATCAGGGAGTAA	TCGGATACTTCAGCGTCAGGA

**(Mouse)**		
***Sm22* (Mouse)**	CAACAAGGGTCCATCCTACGG	ATCTGGGCGGCCTACATCA
***Pcna* (Mouse)**	TTTGAGGCACGCCTGATCC	GGAGACGTGAGACGAGTCCAT
***CyclinD1* (Mouse)**	GCGTACCCTGACACCAATCTC	CTCCTCTTCGCACTTCTGCTC
***Mmp2* (Mouse)**	CAAGTTCCCCGGCGATGTC	TTCTGGTCAAGGTCACCTGTC
***Mmp9* (Mouse)**	CTGGACAGCCAGACACTAAAG	CTCGCGGCAAGTCTTCAGAG
***Gapdh* (Mouse)**	AGGTCGGTGTGAACGGATTTG	TGTAGACCATGTAGTTGAGGTCA

Supplementary Table 2

Used shRNA template sequences

Gene Name	ShRNA oligonucleotide template
Myocd **(Rat)**	5’-GGAAGATCATTCATGTCTT-3’
SRF **(Rat)**	5’-GACCTGCCTCAACTCGCCAGAC-3’
c-Jun **(Rat)**	5’-ACAGGTGGCACAGCTTAAA-3’
P65 **(Rat)**	5’-CCATCAACTTTGATGAGTT-3’
PARP1 **(Rat)**	5’-GGAUGAUCUUCGACGUGGA-3’
